# Supplementary material for: Efficient and selective capture of thorium ions by a covalent organic framework
Source: Nat Commun. 2023 Aug 22;14:5097. doi: 10.1038/s41467-023-40704-0 (PMC10444833; doi:10.1038/s41467-023-40704-0)
Supplement: Supplementary file 1 — Supplementary Information [file 41467_2023_40704_MOESM1_ESM.pdf]

Supplementary Information

Efficient and selective capture of thorium ions by a covalent organic framework

Xiaojuan Liu [1], Feng Gao [1], Tiantian Jin [1], Ke Ma [1], Haijiang Shi [1], Ming Wang [2], Yanan Gao [2], Wenjuan Xue [3], Jing Zhao [1,*], Songtao Xiao [1,*], Yinggen Ouyang [1,*], Guoan Ye [1,*]

[1] Department of Radiochemistry, China Institute of Atomic Energy, Beijing 102413, China.

[2] Key Laboratory of Ministry of Education for Advanced Materials in Tropical Island Resources, Hainan University, Haikou 570228, China.

[3] State Key Laboratory of Separation Membranes and Membrane Processes, Tiangong University, Tianjin 300387, China

* Corresponding authors: Department of Radiochemistry, China Institute of Atomic Energy, Beijing 102413, China (J Zhao, S. Xiao, Y. Ouyang, G. Ye).

E-mail addresses: Jing_zhao@tom.com (Jing Zhao) Songtao_Xiao@tom.com (Songtao Xiao); ouyang_yinggen@163.com (Yinggen Ouyang); guoan_ye@tom.com (Guoan Ye).

Contents

1. Experimental section

2. Supplementary Figures 1-25

3. Supplementary Tables 1-6

4. Supplementary Note 1

5. References

1. Experimental section

1.1 Materials and characterizations

All starting reagents/reactants, except 4,4',4'',4'''-(Pyrene-1,3,6,8-tetrayl) tetraaniline (PyTTA), 2′3′5′6′-tetrafluoro-[1,1′:4′,1″-terphenyl]-4,4′-dicarbaldehyde (TFTDA), 5,6-Bis(4-formylbenzyl)-1-methyl-1*H*-benzimidazole (BFIm), and 5,6-Bis(4-formylbenzyl)-1,3-dimethyl-benzimidazolinium iodide (BFIIm), were purchased commercially. 1,3,6,8-tetrabromopyrene was procured from Yanshen Technology Co., Ltd. 4-formylbenzeneboronic acid pinacol ester, camphorsulfonic, and 4,7-dibromo-2,1,3-benzothiadiazole were procured from Shanghai Adamas Reagent Co., Ltd. Tetrakis (triphenylphosphine) palladium (0), triethyl orthoformate, and 4-aminophenylboronic acid pinacol ester were procured from Meryer (Shanghai) Chemical Technology Co., Ltd. Methyl iodide was procured from Xiya Reagent. 1,4-dibromotetrafluorobenzene was procured from J&K Scientific. Sodium borohydride, anhydrous $MgSO_4$, anhydrous K_2CO_3, dioxane, ethyl acetate, tetrahydrofuran, ethanol, dichloromethane, and methanol were procured from Greagent of Shanghai Titan Scientific Co., Ltd. All reagents are of analytical grade and used directly as received without further purification.

Powder X-ray diffraction (PXRD) patterns analyses of the adsorbents in the form of powder to characterize the crystal structure were carried out by Bruker AXS X-ray diffractometer equipped with Nifiltered Cu KR radiation (40 kV, 40 mA). The patterns were recorded over the range of 2θ angles from 2-30 ° in steps of 0.02 °/s. PXRD patterns of Th(IV)-loaded samples were carried out on Rigaku Ultima IV. Fourier

transform infrared spectroscopy (FTIR) spectra were obtained on Nicolet iS50 spectrometer (Thermo Fisher Scientific Company) by scans of 32 with a resolution of 4 cm^{-1} by the KBr pellet method. The range of wavenumbers is 4000-400 cm^{-1}. X-ray photoelectron spectroscopy (XPS) spectra were acquired using Perkin-Elmer PHI 5000CESCA system equipped with Al Kα radiation (1486.6 eV). The assignment of the peak was based on the Handbook of X-ray Photoelectron Spectroscopy. ^{1}H and ^{13}C nuclear magnetic resonance (NMR) spectra were recorded on a Bruker Advance III 400 MHz NMR spectrometer. High-resolution mass spectral (HRMS) data was recorded by SHIMADZU LCMS-IT-TOF. Gas adsorption isotherms were measured at 77 K using a liquid N_2 bath on a surface area analyzer, Autosorb iQ. The fresh sample was activated in the degassing station of the instrument at 30 °C for 2 h, and then at 120 °C for 13 h to make the pores guest-free. Thermogravimetric analysis (TGA) was recorded using a Mettler Toledo DSC3+ thermogravimetric analyzer over the temperature range of 25-900 ℃ in a nitrogen atmosphere with a heating rate of 10 ℃ min^{-1}. Scanning electron microscopy (SEM) and energy dispersive spectrometer (EDS) were collected using a Hitachi S-4800. The concentrations of all metal ions used in this work were analyzed by ICP-MS (Optima 8000, PerkinElmer) using the standard curve method.

1.2 Preparation of the Py-TFImI-25 COF and Py-TFIm-25 COF

1.2.1 Synthesis of 4,4',4'',4'''-(Pyrene-1,3,6,8-tetrayl) tetraaniline (PyTTA)

The synthesis process of the organic building block PyTTA was referred to the literature[1,2] and improved, and the specific synthetic pathway is shown in Supplementary Fig. 1, which was mainly prepared from 1,3,6,8-tetrabromopyrene with

4-aminophenylboronic acid pinacol ester by Suzuki coupling reaction.

K_2CO_3 (2.18 g, 15.7 mmol), 1,3,6,8-tetrabromopyrene (1.48 g, 2.86 mmol), 4-aminophenylboronic acid pinacol ester (3.01 g, 13.7 mmol), and tetrakis (triphenylphosphine) palladium (0) (0.33 g, 0.29 mmol) were placed into a three-necked round-bottomed flask. 8 mL of deoxygenated ultrapure water and 32 mL of dry dioxane were added under N_2 atmosphere, respectively, and the reaction was refluxed at 85 °C for 72 h. After the reaction, the product was cooled to room temperature, and the solid on the filter paper was repeatedly washed with water until the filtrate was colorless. The solid was then dispersed in methanol and sonicated, suction filtered, the solid on the filter paper was washed several times with methanol, and the solid PyTTA was collected, and vacuum-dried at room temperature overnight. The yield was 74%. ^{1}H NMR (400 MHz, DMSO) δ 8.14 (s, 4H), 7.80 (s, 2H), 7.35 (d, *J* = 8.3 Hz, 8H), 6.78 (d, *J* = 8.4 Hz, 8H), 5.32 (s, 8H).

1.2.2 Synthesis of 2′3′5′6′-tetrafluoro-[1,1′:4′,1″-terphenyl]-4,4′-dicarbaldehyde (TFTDA)

TFTDA was synthesized[2] by a Suzuki coupling reaction similar to PyTTA (see Supplementary Fig. 2). K_2CO_3 (2.63 g, 19.0 mmol), 1,4-dibromotetrafluorobenzene (1.65 g, 5.36 mmol), 4-formylbenzeneboronic acid pinacol ester (3.36 g, 14.48 mmol) and tetrakis(triphenylphosphine) palladium(0) (0.35 g, 0.30 mmol) were added into the three-necked round-bottomed flask. 12 mL of deoxygenated ultrapure water and 50 mL of dry dioxane were added under N_2 atmosphere, respectively, and the reaction was refluxed at 100 °C for 24 h. Then the reaction product was cooled to room temperature,

the obtained solid was suction filtered, washed with a small amount of water, ethyl acetate, and ethanol in turn, and the product was dried at 80°C overnight to obtain TFTDA as a white light solid powder. The yield was 80%. ^{1}H NMR (400 MHz, DMSO-d_6) δ 10.12 (s, 2H), 8.12 (d, J = 8.4 Hz, 4H), 7.85 (d, J = 8.0 Hz, 4H).

1.2.3 Synthesis of 5,6-Bis(4-formylbenzyl)-1-methyl-1*H*-benzimidazole (BFIm)

The synthesis process of the organic building unit BFIm was referred to the literature[2-4] and improved, and the specific synthesis pathway is shown in Supplementary Fig. 3.

4,7-dibromo-2,1,3-benzothiadiazole (3.50 g, 11.91 mmol) was added to the two-necked round-bottomed flask, and under N_2 atmosphere, 400 mL of absolute ethanol and 40 mL of dry tetrahydrofuran (THF) were added for stirring. After the reaction system was cooled to 0 °C, sodium borohydride (8.11 g, 211.31 mmol) was added, and then transferred to room temperature for 3 h. After the reaction, it was placed in an ice-water bath, quenched by slowly adding 100 mL of deionized water, and rotary-evaporated under reduced pressure. The crude product was dissolved in 150 mL of ether, poured into a separatory funnel, and washed with saturated brine three times, the organic phase was dried over anhydrous $MgSO_4$, filtered, and rotary-evaporated under reduced pressure to obtain an orange flaky solid 4,5-Dibromo-1,2-benzenediamine (P1). To prevent the decomposition of P1, it needs to be protected with N_2 or used directly in the next reaction step. The yield was 86%. ^{1}H NMR (400 MHz, $CDCl_3$) δ 6.85 (s, 2H), 3.74 (s, 4H).

P1 (2.16 g, 8.11 mmol) was dissolved in 90 mL of CH_2Cl_2, then triethyl orthoformate

(1.35 mL, 12.39 mmol) was added, and after stirring at room temperature for 10 min, 27.5 mg of camphorsulfonic acid was added to the reaction system and the stirring was continued for 3 h. A large amount of white solid was produced, which was filtered and washed several times with a small amount of diethyl ether to obtain a white layered solid 5,6-Dibromobenzimidazole (P2). The yield was 67%. ^{1}H NMR (400 MHz, DMSO-d_6) δ 8.41 (s, 1H), 7.38 (s, 2H).

P2 (1.97 g, 7.14 mmol) and K_2CO_3 (2.96 g, 21.4 mmol) were added to the three-necked flask, then 50 mL of absolute ethanol was added, the temperature was raised to 50 °C, and then 1.0 mL of methyl iodide was added dropwise. The reaction system was sealed and refluxed for 3 h. After the reaction was completed, the reaction product was cooled to room temperature, 20 mL of deionized water was added, and rotary evaporated under reduced pressure to remove ethanol, filtered and washed with water and then with n-hexane/ethyl ether (1:1), and vacuum-dried at 60 °C overnight to obtain beige solid powder 5,6-Dibromo-1-methyl-1*H*-Benzimidazole (P3). The yield was 86%. ^{1}H NMR (400 MHz, DMSO-d_6) δ 8.36 (s, 1H), 7.37 (s, 2H), 4.09 (s, 3H).

K_2CO_3 (2.76 g, 20 mmol), P3 (1.93 g, 6.67 mmol), 4-formylbenzeneboronic acid pinacol ester (4.67 g, 15.35mmol), and 385 mg tetrakis(triphenylphosphine) palladium(0) (385 mg, 0.33 mmol) were added to the three-necked round-bottomed flask, respectively, and deoxygenated deionization was added under N_2 atmosphere. 12 mL of water and 50 mL of dry dioxane. The reaction was refluxed at 100 °C for 15 h. After the reaction system was cooled to room temperature, deionized water was added, and the mixture was poured into a separatory funnel. After the aqueous phase was

extracted with ethyl acetate, it was combined with the organic phase and washed with saturated NaCl solution. The product was rotary evaporated under reduced pressure, and the crude product was washed with n-hexane/diethyl ether (2/1) to obtain a brown powdery solid 5,6-Bis(4-formylbenzyl)-1-methyl-1*H*-benzimidazole (P4). The yield was 65%. ^{1}H NMR (400 MHz, $CDCl_3$) δ 10.14 (s, 1H), 10.09 (s, 1H), 8.21 (d, *J* = 8.1 Hz, 2H), 8.03 (d, *J* = 11.6 Hz, 4H), 7.93 (s, 1H), 7.67 (d, *J* = 8.0 Hz, 2H), 7.54 (d, *J* = 7.7 Hz, 1H), 7.27 (s, 1H), 3.46 (s, 3H). ^{13}C NMR (101 MHz, $CDCl_3$) δ 192.28 (s), 191.83 (s), 145.89 (s), 144.70 (s), 144.44 (s), 142.52 (s), 135.89 (s), 135.37 (s), 132.44 (s), 131.55 (s), 130.70 (s), 130.07 (s), 129.99 (s), 129.54 (s), 126.06 (s), 125.26 (s), 121.93 (s), 34.71 (s). HRMS-ESI (m/z) $[M+H]^+$ Calcd for $C_{22}H_{17}N_2O_2^+$, 341.1212, Found 341.1262.

1.2.4 Synthesis of 5,6-Bis(4-formylbenzyl)-1,3-dimethyl-benzimidazolinium iodide (BFIIm)

The synthesis process of the organic building unit BFIIm was referred to the literature[2,5] and improved. As shown in Supplementary Fig. 4, P4 (0.40 g, 1.17 mmol), 12 mL of acetonitrile, and methyl iodide (0.73 mL, 11.7 mmol) were added to a 100 mL single-neck flask and refluxed at 85 °C for 24 h. It was cooled to room temperature, evaporated under reduced pressure, and the solid was washed with n-hexane/ethyl acetate (2/1) and dried under vacuum at 100 °C overnight. The brown product P5 was obtained. The yield was 65%. ^{1}H NMR (400 MHz, DMSO-d_6) δ 10.17 (s, 2H), 9.72 (s, 1H), 8.12 (d, *J* = 8.1 Hz, 4H), 7.82 (d, *J* = 8.0 Hz, 4H), 7.63 (s, 2H), 3.56 (s, 6H).

1.2.5 Synthesis of Py-TFImI-25 COF and Py-TFIm-25 COF

PyTTA (22.7 mg, 0.04 mmol), TFTDA (21.5 mg, 0.06 mmol), and BFIIm (9.6 mg, 0.02 mmol) (or BFIm with 6.9 mg, 0.02 mmol) were added to a 10 mL Pyrex tube. Add 2.0 mL of o-dichlorobenzene and 0.2 mL of 6 M AcOH, respectively, and mix well by sonication. The solvent-containing part at the lower end of the Pyrex tube was refrigerated and degassed in 77 K liquid nitrogen, circulated about three times, and the tube was sealed at a high temperature. After the system was completely restored to room temperature, it was transferred to a constant temperature oven at 120 °C and left to react for 72 h. After the reaction, the product was fully washed with dry THF 5 times, then soaked in fresh 30 mL THF solution for about 8 h. Then, the products were filtered and washed three times with dry acetone. Vacuum dry at 20 °C overnight to obtain Py-TFImI-25 COF (or Py-TFIm-25 COF). The yield was 82% for Py-TFImI-25 COF, and 80% for Py-TFIm-25 COF.

1.3 Thorium ions capture

1.3.1 Effect of pH on the Th(IV) adsorption

Five sets of ~25 mg L^{-1} Th(IV) solutions were prepared at 50 mL each. The pH value was adjusted to 1.0, 2.0, 3.0, 4.0, and 5.0 by adding 0.1 mol L^{-1} NaOH and 0.1 mol L^{-1} HNO_3 by dropwise, respectively. 4.5 mL of the Th(IV) solution was mixed with 1.5 mg COF material at 25 °C and shaking with 200 rpm for 1 h. The adsorbed solution was filtered through a 0.22 μm aqueous nylon filter, and the Th(IV) concentration before and after adsorption was measured. The following equations were used to obtain the equilibrium adsorption capacity q_e (mg g^{-1}), the distribution coefficient K_d (mL g^{-1}), and separation factor *SF* (a. u.).

$$q_{\mathrm{e}} = \frac{C_0 - C_{\mathrm{e}}}{m} \times V \quad (1)$$

$$K_{\mathrm{d}} = \frac{C_0 - C_{\mathrm{e}}}{C_{\mathrm{e}} m} \times V \quad (2)$$

$$SF_{\mathrm{a/b}} = \frac{K_{\mathrm{d}}^{\mathrm{a}}}{K_{\mathrm{d}}^{\mathrm{b}}} \quad (3)$$

Where, C_0, C_e, V, m refer to the initial concentration, equilibrium concentration, solution volume, and adsorbent mass of an element, respectively. a, and b refer to two different elements, respectively.

1.3.2 Adsorption kinetics

The kinetic studies were carried out under the following conditions. The initial concentration of Th(IV) solution was around 40 mg L^{-1} with a pH value of 4, and the solid-liquid ratio was maintained at 1:3000 g mL^{-1} by adding 1.5 mg COF materials into 4.5 mL Th(IV) solution. At each predetermined time, a small amount of supernatant was taken and filtered through a 0.22 aμm aqueous nylon filter to test the Th(IV) solution concentration before and after adsorption. The adsorption amount (q_t, mg g^{-1}) at a contact time of t was calculated by Equation (4).

$$q_{\mathrm{t}} = \frac{C_0 - C_{\mathrm{t}}}{m} \times V \quad (4)$$

The adsorption kinetic data was analyzed using two models, the pseudo-first-order model and the pseudo-second-order model, and the linearized forms of the two models are shown in Equation (5) and Equation (6), respectively.

$$\ln(q_{\mathrm{e}} - q_{\mathrm{t}}) = \ln q_{\mathrm{e}} - k_1 t \quad (5)$$

$$\frac{t}{q_{\mathrm{t}}} = \frac{1}{k_2 q_{\mathrm{e}}^2} + \frac{t}{q_{\mathrm{e}}} \quad (6)$$

Where q_e and q_t (mg g^{-1}) are the amounts of adsorbates adsorbed at equilibrium and

the predetermined time t (min), q_e (mg g^{-1}) is fitting equilibrium capacity. k_1 (min^{-1}) and k_2 ($g.mg^{-1}.min^{-1}$) are the pseudo-first-order and pseudo-second-order adsorption rate constant, respectively.

1.3.3 Effect of different Th(IV) concentration on the capture capacity

The initial concentration of Th(IV) solution was ranging from 15 to 300 mg L^{-1} with a pH value of 4, and the solid-liquid ratio was maintained at 1:3000 g mL^{-1} by adding 1.5 mg COF materials into 4.5 mL Th(IV) solution. At various metal ion concentrations, the saturation adsorption capabilities of the adsorbents toward Th(IV) were measured.

1.3.4 Adsorption selectivity

A multi-ion solution of Th(IV), U(VI), Sr(II), Cs(I), La(III), Pr(III), Nd(III), Sm(III), Eu(III), and Gd(III) was prepared with each metal ion maintaining at a concentration of around 25 mg L^{-1} and the pH was adjusted to 4. The solid-liquid ratio was 1:3000 g mL^{-1} by adding 1.5 mg COF materials into 4.5 mL multi-ion solution. The batch adsorption experiments were carried out at a temperature of 25 °C and a speed of 200 rpm, and then the multi-ion solution after adsorption was separated with a 0.22 μm aqueous nylon filter to measure the concentration of Th(IV) and the competing ions.

1.4 Computational Details

In this work, the crystal models of Py-TFImI-25 and Py-TFIm-25 COF were constructed with the Crystal Building module of the Material Studio software. Then, a series of geometry optimizations were performed with UFF force field at the ultra-fine quality in the Forcite module, allowing optimization of all lattice parameters and atomic coordinate. All the optimized COF materials were used for subsequent simulation of

the powder diffraction patterns with the Reflex module in the Material Studio software. Both the eclipsed AA and staggered AB stacking modes were tried for the Py-TFImI-25 and Py-TFIm-25 synthesized here. Compared the experimental XRD results with the simulated XRD, finally, the eclipsed AA stacking modes were settled due to the good match of the experimental XRD results with the simulated XRD on the eclipsed AA stacking mode. Based on the obtained results, periodic density functional theory (DFT) calculations were performed with the cell parameters of 42.7443 × 15.8124 × 49.7048 $Å^3$, α=87.9005°, β= 89.7684°, γ= 81.1063° for Py-TFImI-25 COF and 45.3198 × 15.8606 × 48.2641 $Å^3$, α=89.3474°, β= 90.1480°, γ= 85.4182° Py-TFIm-25 COF, respectively. During the structural DFT optimization, all the atoms in the periodic Py-TFImI-25 COF, Py-TFIm-25 COF, and the adsorbed heavy metal ions were allowed to be fully relaxed. The Pawley refinement was performed to iteratively optimize the lattice parameters until the R_{WP} value converges and the observed profile fitted well with the refined one. Detailed results are in Supplementary Table 1 and 2.

Structure optimization and energy calculations were performed using density functional theory, as implemented in the CP2K code of the QUICKSTEP program by employing a mixed Gaussian and plane-wave basis sets[6-8]. Core electrons were represented with norm-conserving Goedecker-Teter-Hutter pseudopotentials[6,9,10], and the valence electron wave function was expanded in a double-zeta basis set with polarization functions[11] along with an auxiliary plane wave basis set with an energy cutoff of 400 Ry. The generalized gradient approximation exchange-correlation functional of Perdew, Burke, and Enzerhof (PBE)[12] was used. Each configuration was

optimized with the Broyden-Fletcher-Goldfarb-Shanno (BGFS) algorithm with SCF convergence criteria of 1.0×10^{-8} au. To compensate the long-range van der Waals dispersion interaction between the adsorbate and the framework, the DFT-D3 scheme[13] with an empirical damped potential term was added into the energies obtained from exchange-correlation functional in all calculations. The self-consistent continuum solvation (SCCS) model with a dielectric constant of 78.54 was used to simulate the solvent environment[14-16].

The adsorption potential energy between the heavy metal cations M (M = Th^{4+}, La^{3+}, Nd^{3+}, UO_2^{2+}, Sr^{2+}, and Cs^{+}) and the Py-TFImI-25 COF and Py-TFIm-25 COF substrates can be calculated using the following equation:

$$\Delta E_{\mathrm{ads}} = E_{\mathrm{M@substrate}} - E_{\mathrm{substrate}} - E_{\mathrm{M}} \tag{7}$$

In Equation (7), $E_{\mathrm{M@substrate}}$ and $E_{\mathrm{substrate}}$ represent the total energies of the substrate with and without adsorbate, respectively. E_{M} is the total energy of the metal cation *M*. According to this equation, negative adsorption energy corresponds a stable adsorption structure.

2. Supplementary Figures 1-25

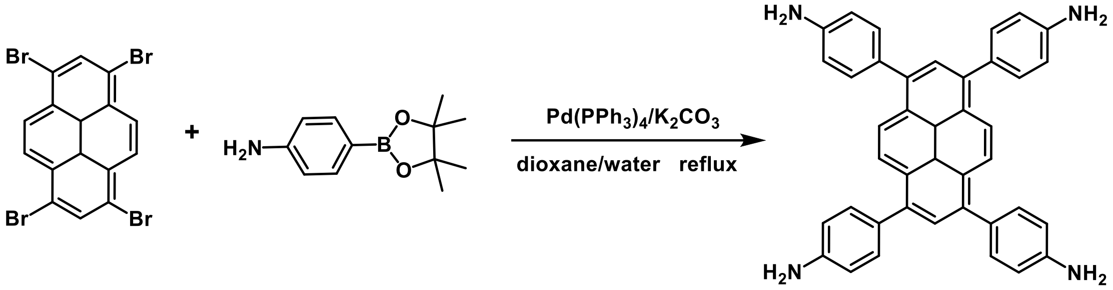

Supplementary Fig. 1. Synthesis of starting material. Synthetic route to 4,4',4'',4'''-(Pyrene-1,3,6,8-tetrayl) tetraaniline (PyTTA).

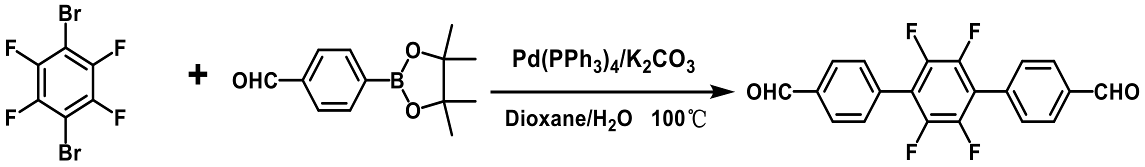

Supplementary Fig. 2. Synthesis of starting material. Synthetic route to 2′3′5′6′-tetrafluoro-[1,1′:4′,1″-terphenyl]-4,4′-dicarbaldehyde (TFTDA).

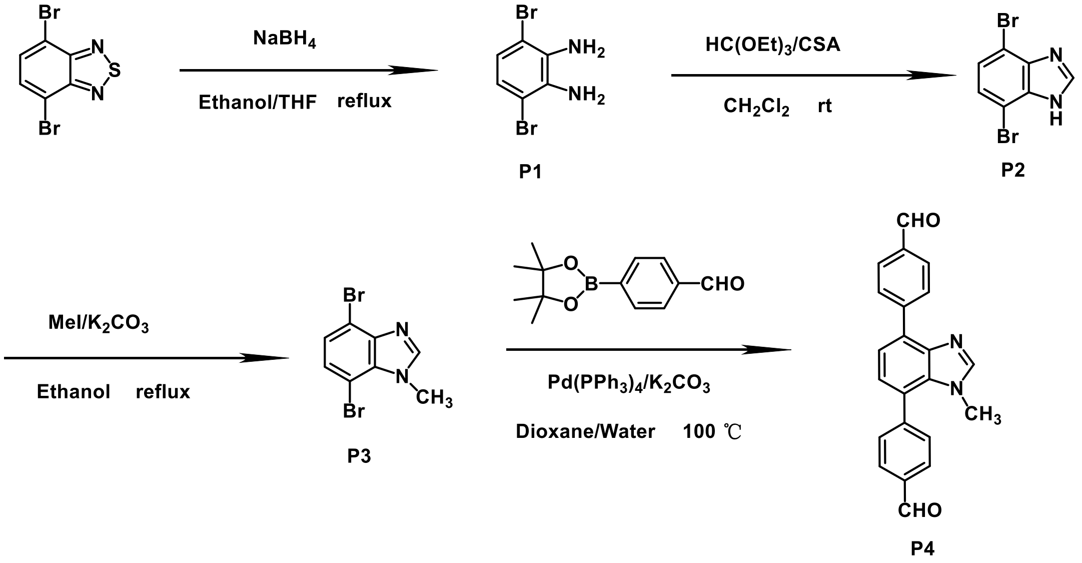

Supplementary Fig. 3. Synthesis of starting material. Synthetic route to 5,6-Bis(4-formylbenzyl)-1-methyl-1*H*-benzimidazole (BFIm, P4).

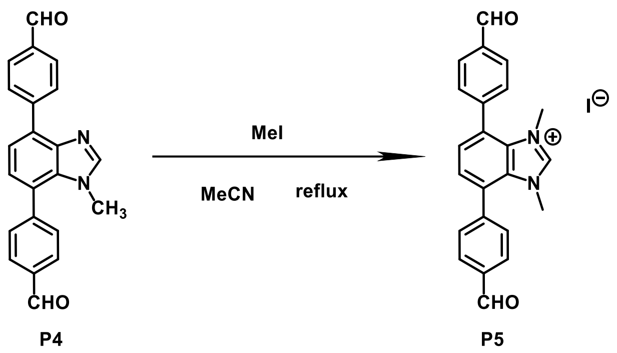

Supplementary Fig. 4. Synthesis of starting material. Synthetic route for the preparation of 5,6-Bis(4-formylbenzyl)-1,3-dimethyl-benzimidazolinium iodide (BFIIm, P5).

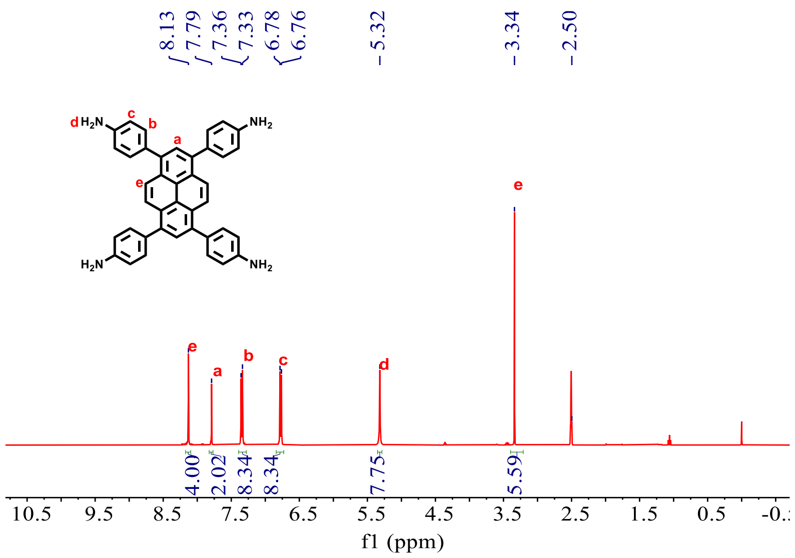

Supplementary Fig. 5. Characterization of starting material. ^{1}H NMR spectrum of 4,4',4'',4'''-(Pyrene-1,3,6,8-tetrayl) tetraaniline (PyTTA).

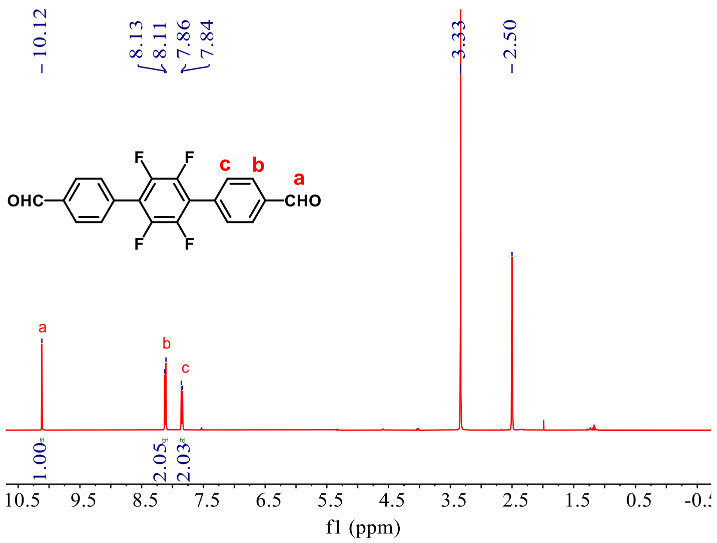

Supplementary Fig. 6. Characterization of starting material. ^{1}H NMR spectrum of 2′3′5′6′-tetrafluoro-[1,1′:4′,1″-terphenyl]-4,4′-dicarbaldehyde (TFTDA).

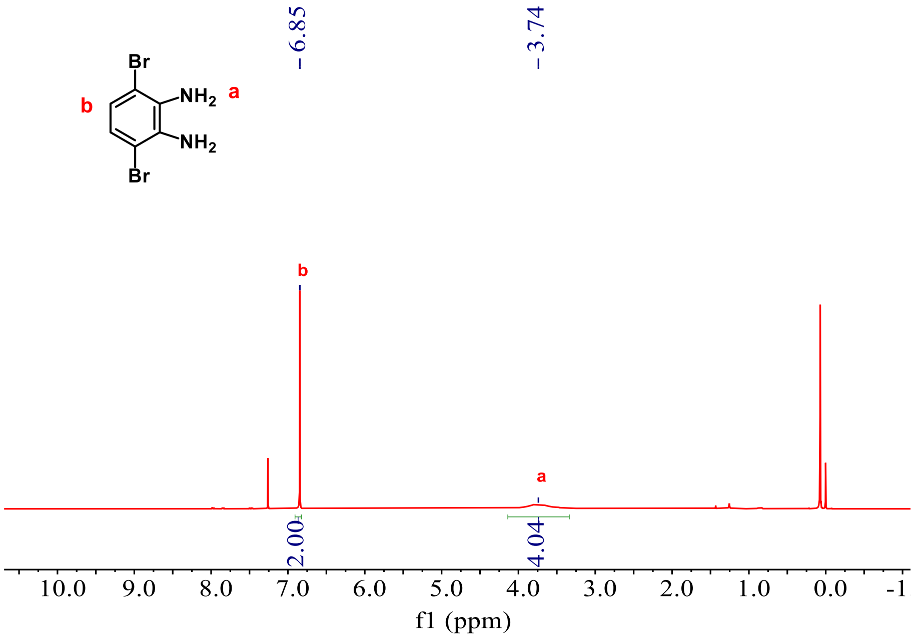

Supplementary Fig. 7. Characterization of starting material. ^{1}H NMR spectrum of 4,5-Dibromo-1,2-benzenediamine (P1).

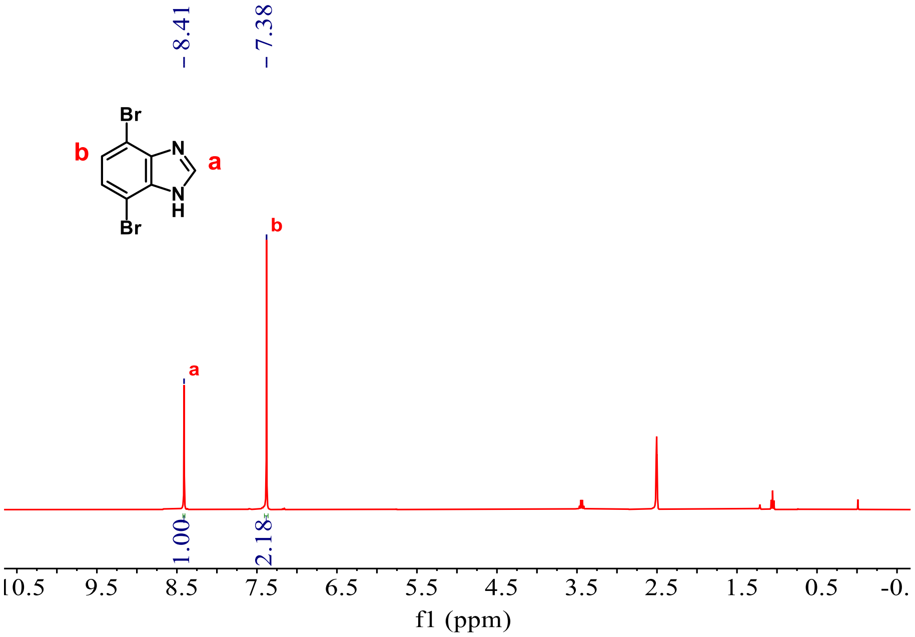

Supplementary Fig. 8. Characterization of starting material. ^{1}H NMR spectrum of 5,6-Dibromobenzimidazole (P2).

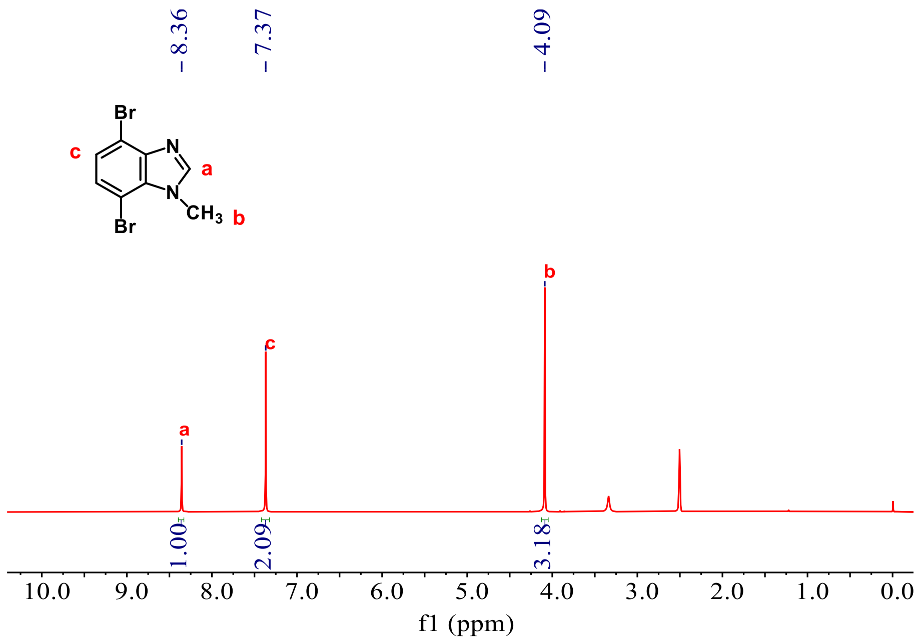

Supplementary Fig. 9. Characterization of starting material. ^{1}H NMR spectrum of 5,6-Dibromo-1-methyl-1*H*-Benzimidazole (P3).

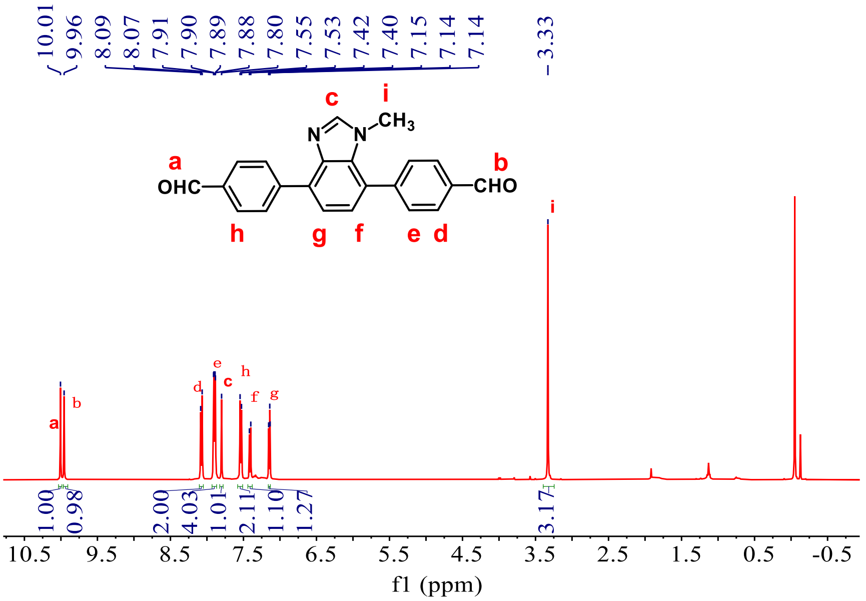

Supplementary Fig. 10. Characterization of starting material. ^{1}H NMR spectrum of 5,6-Bis(4-formylbenzyl)-1-methyl-1*H*-benzimidazole (BFIm, P4).

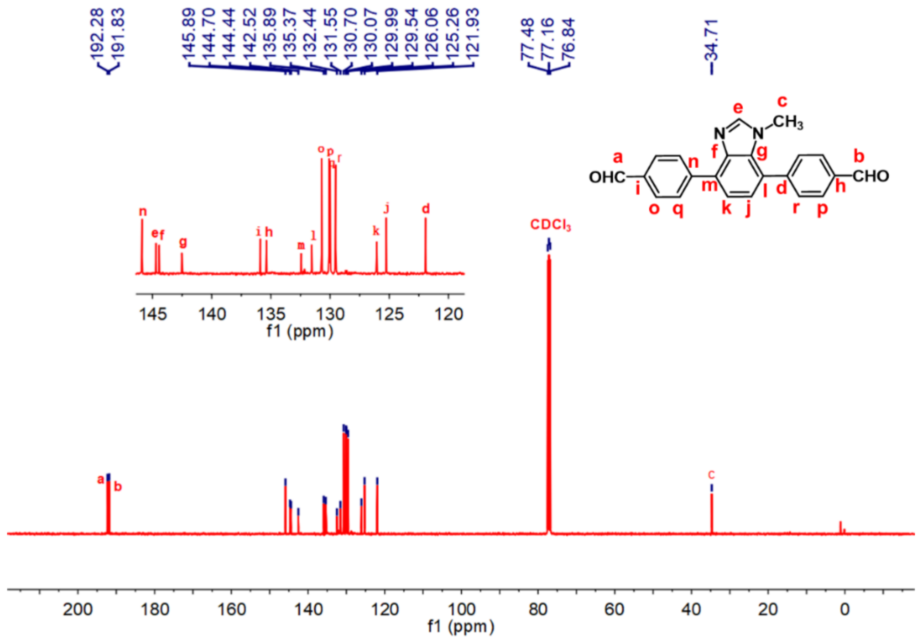

Supplementary Fig. 11. Characterization of starting material. ^{13}C NMR spectrum of 5,6-Bis(4-formylbenzyl)-1-methyl-1*H*-benzimidazole (BFIm, P4).

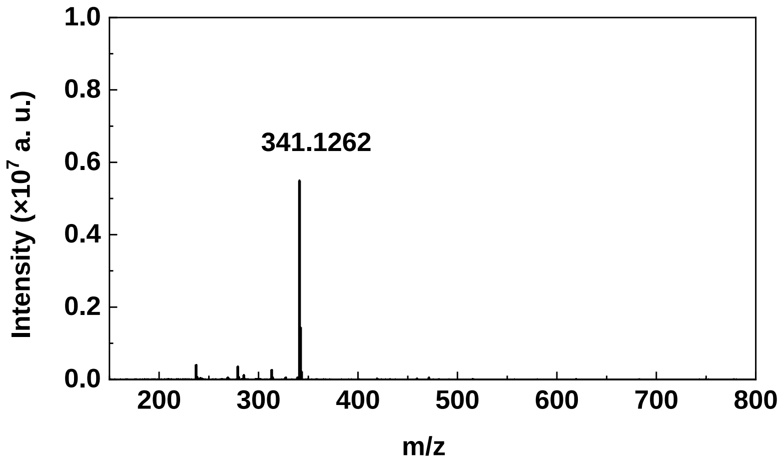

Supplementary Fig. 12. Characterization of starting material. HRMS data of 5,6-Bis(4-formylbenzyl)-1-methyl-1*H*-benzimidazole (BFIm, P4).

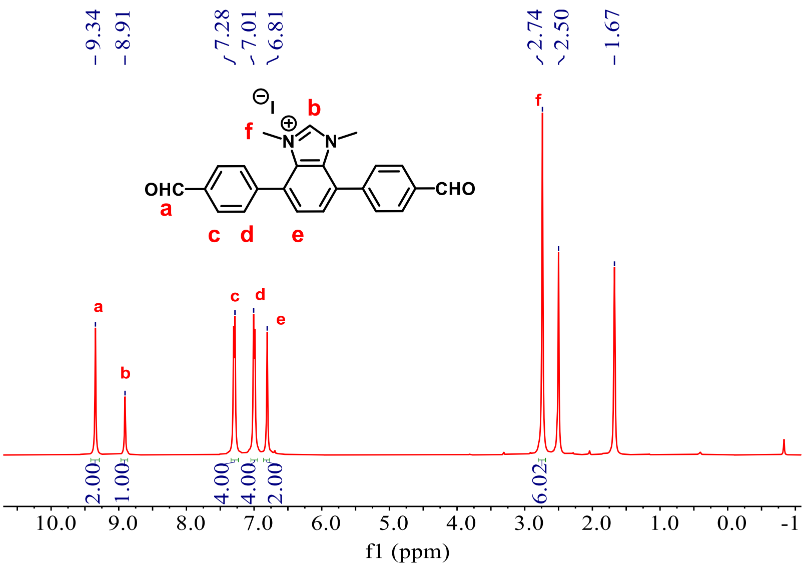

Supplementary Fig. 13. Characterization of starting material. ^{1}H NMR spectrum of 5,6-Bis(4-formylbenzyl)-1,3-dimethyl-benzimidazolinium iodide (BFIIm, P5).

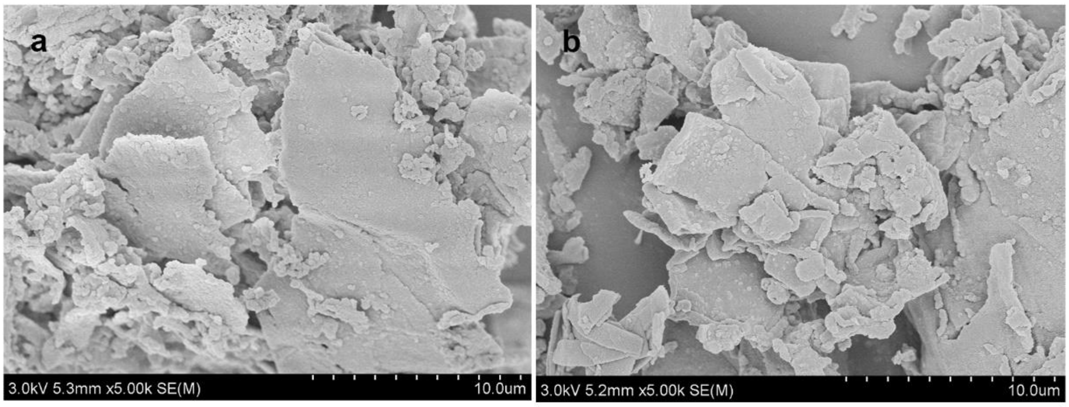

Supplementary Fig. 14. COF characterization. SEM images of **a** Py-TFImI-25 COF and **b** Py-TFIm-25 COF.

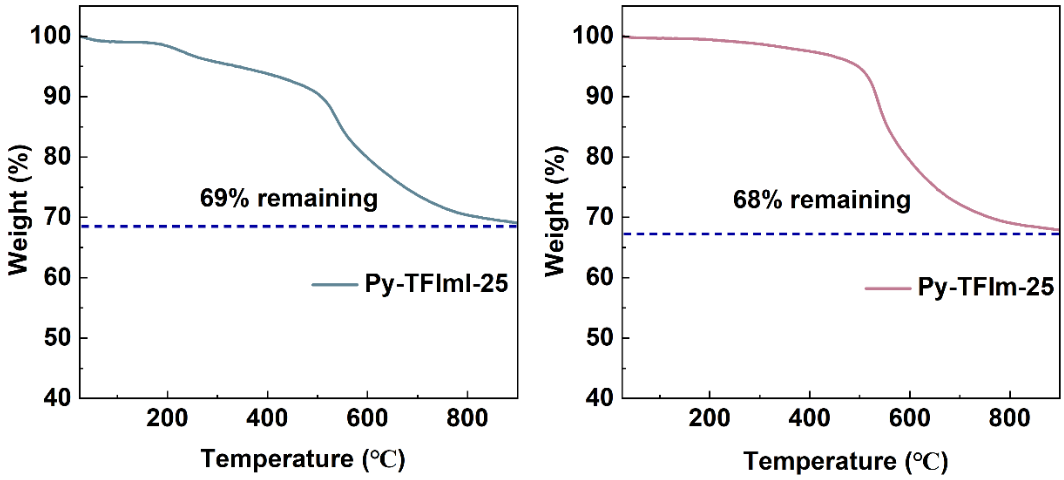

Supplementary Fig. 15. COF characterization. TGA measurements of Py-TFImI-25 COF and Py-TFIm-25 COF.

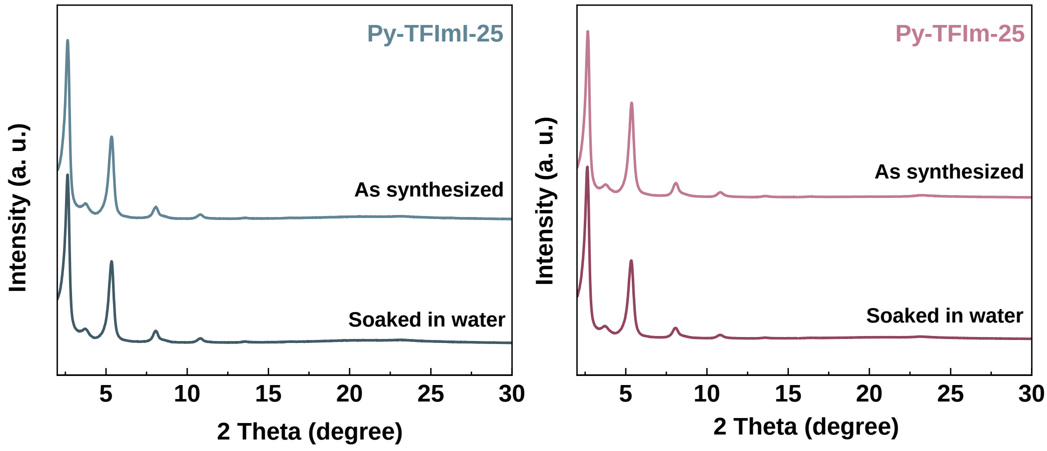

Supplementary Fig. 16. COF characterization. PXRD patterns of Py-TFImI-25 COF and Py-TFIm-25 COF after soaking in water. (Solid-liquid ratio = 1:3000 g mL^{-1}, Shaking time = 1 h, T= 25℃).

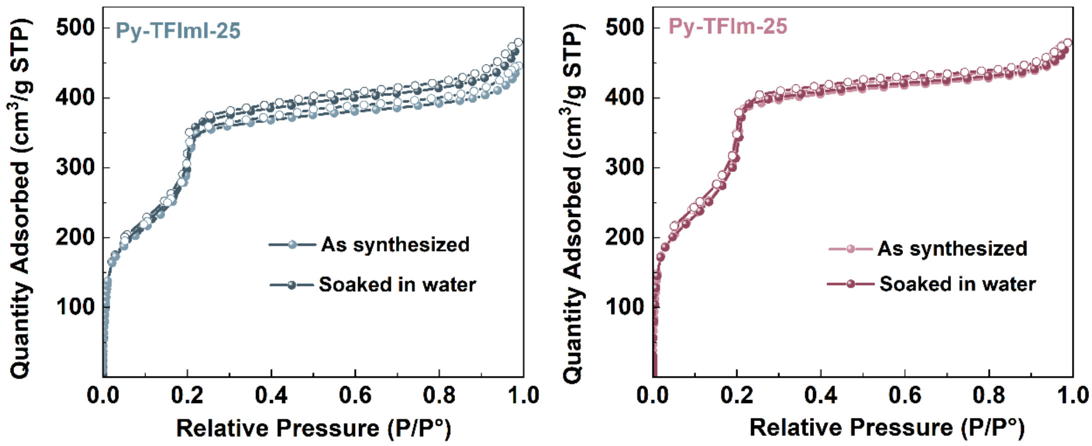

Supplementary Fig. 17. COF characterization. N_2 sorption isotherms of Py-TFImI-25 COF and Py-TFIm-25 COF after soaking in water. (Solid-liquid ratio = 1:3000 g mL^{-1}, Shaking time = 1 h, T= 25℃).

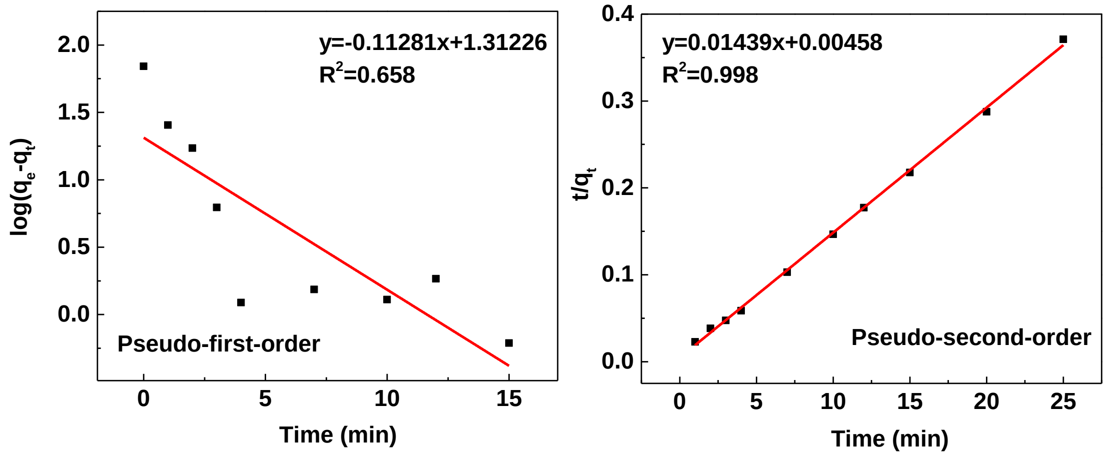

Supplementary Fig. 18. Adsorption performance. Linear fitting of Py-TFImI-25 COF to Th(IV) adsorption kinetics by two kinetic models.

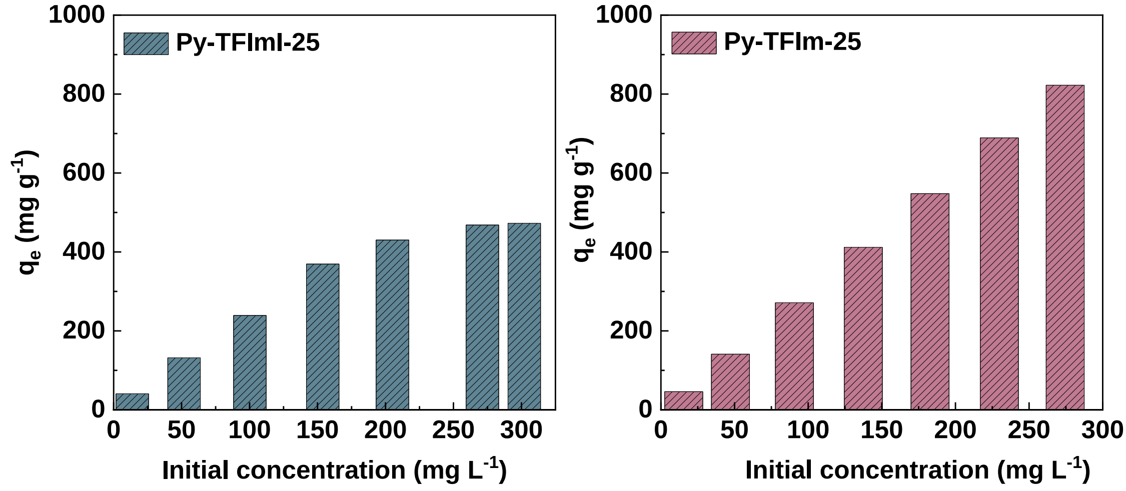

Supplementary Fig. 19. Adsorption performance. Effect of different Th(IV) concentration on the capture capacity of Py-TFImI-25 COF and Py-TFIm-25 COF.

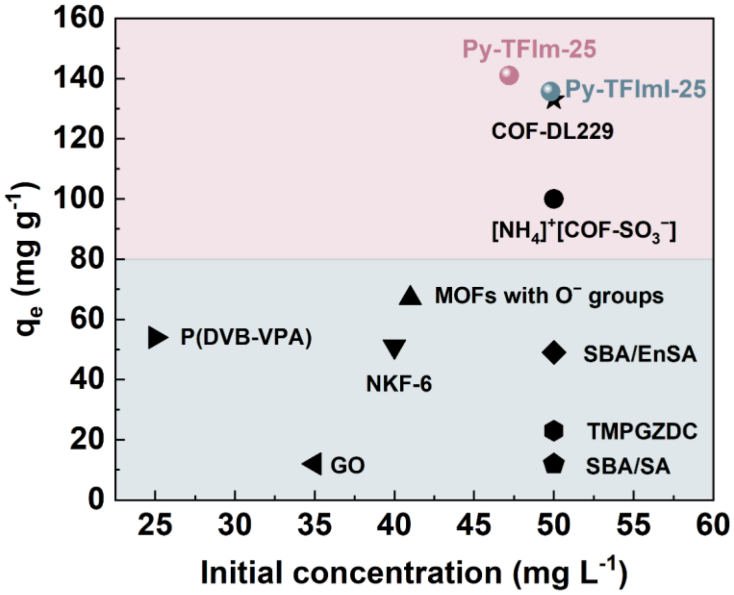

Supplementary Fig. 20. Adsorption performance. Comparison of Th(IV) uptake capacity of prepared COF materials with the various materials for Th(IV) capture.

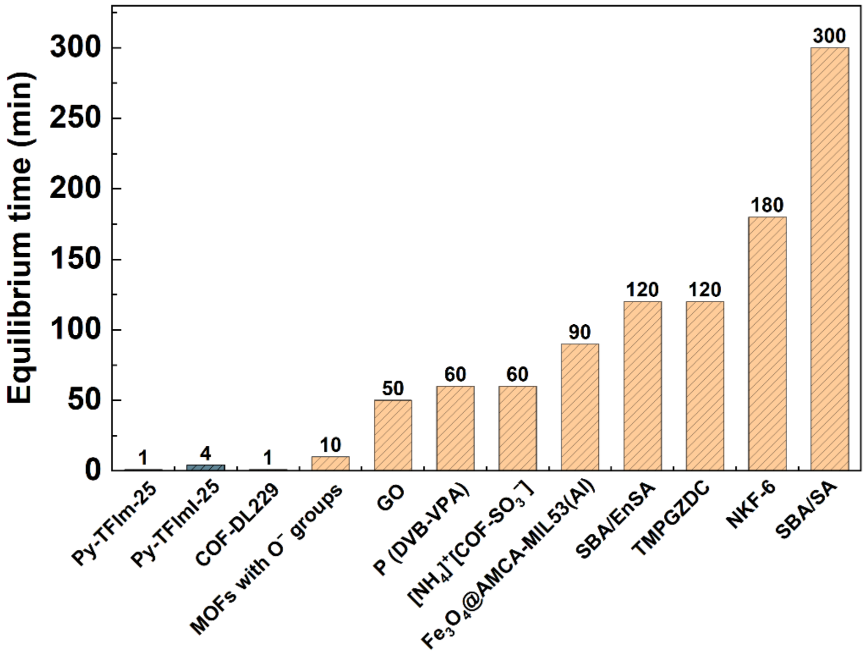

Supplementary Fig. 21. Adsorption performance. Comparison of the equilibrium time of prepared COF materials with the various materials for Th(IV) capture.

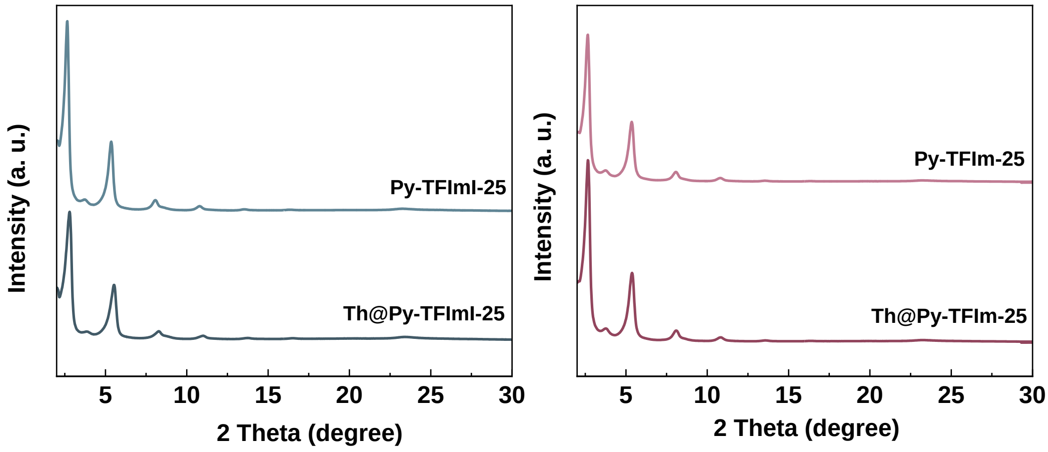

Supplementary Fig. 22. COF characterization. PXRD patterns of Py-TFImI-25 COF and Py-TFIm-25 COF before and after Th(IV) adsorption.

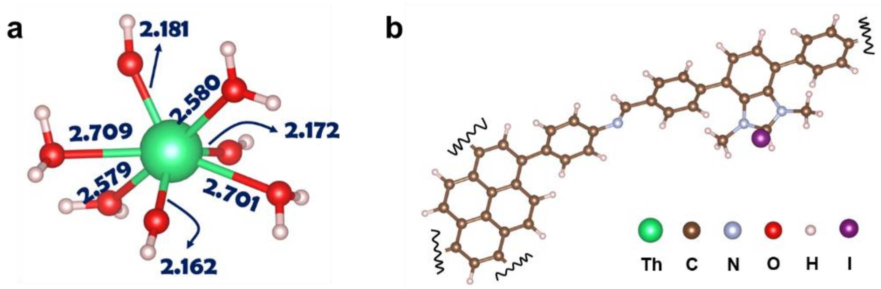

Supplementary Fig. 23. DFT calculations. The optimized structures of the most energetically stable of **a** $[Th(OH)_3(H_2O)_4]^+$ and **b** Py-TFImI-25 COF framework.

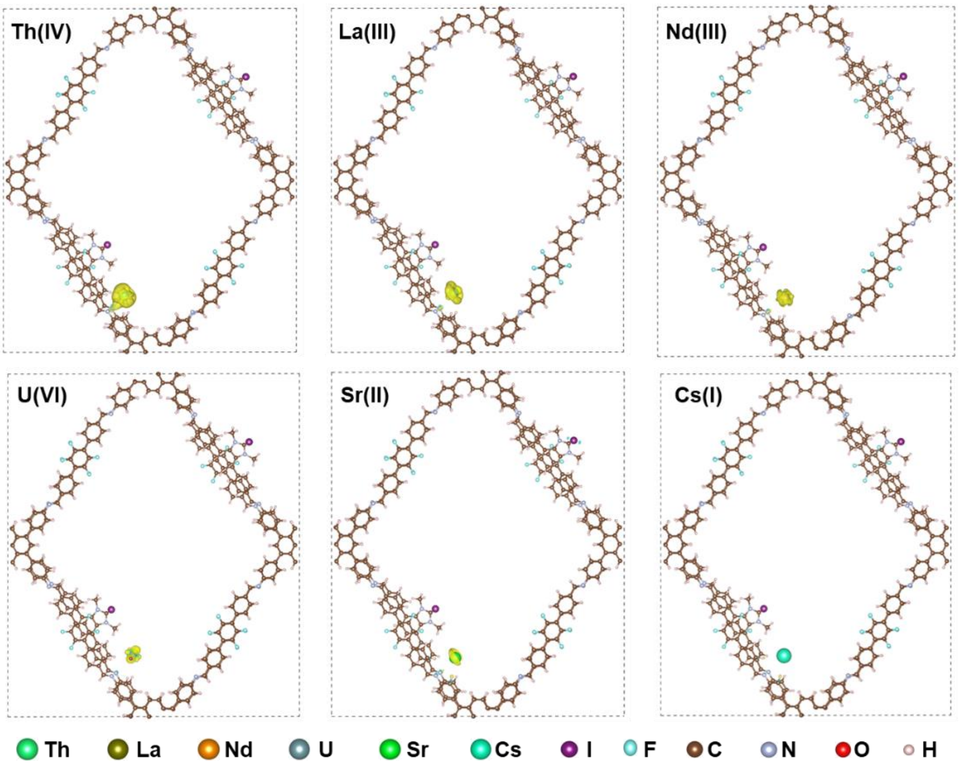

Supplementary Fig. 24. DFT calculations. Adsorption configuration of Py-TFImI-25 COF towards different ions.

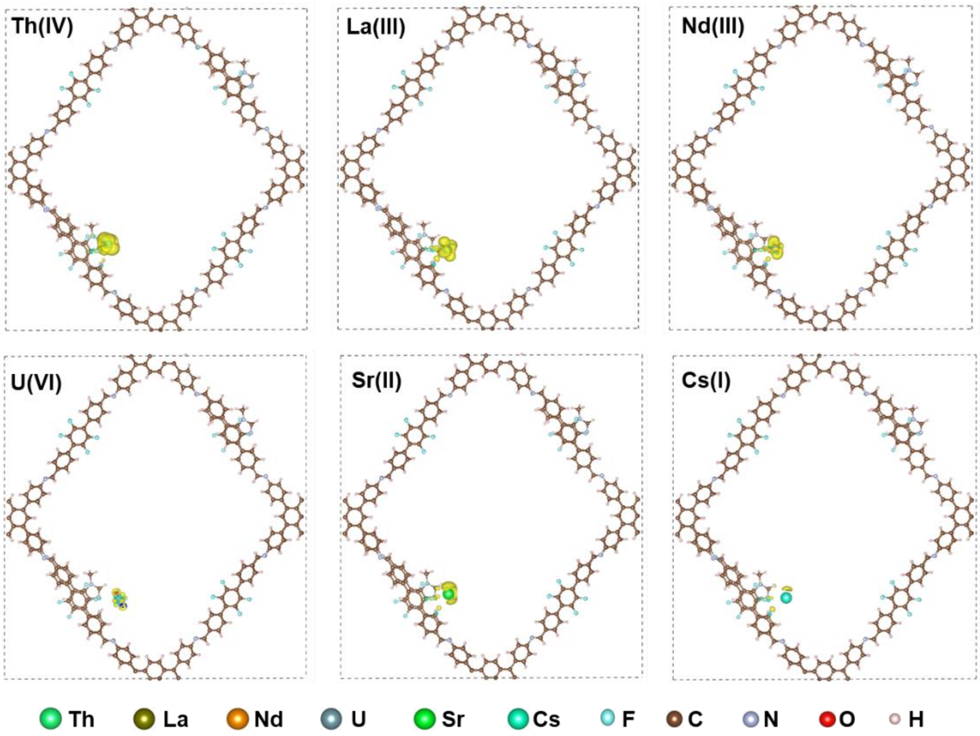

Supplementary Fig. 25. DFT calculations. Adsorption configuration of Py-TFIm-25 COF towards different ions.

3. Supplementary Tables 1-6

Supplementary Table 1. Fractional atomic coordinates in the refined unit cell of Py-TFImI-25 COF

Py-TFImI-25 COF: Space group: P1							
a = 43.30 Å, b = 7.94 Å, c = 50.22 Å							
α = 87.41°, β = 90.59°, and γ = 81.35°							
Atom	x	y	z	Atom	x	y	z
H1	0.27703	0.37074	0.65689	H280	0.74545	0.97106	0.17251
H2	0.30721	0.20104	0.64569	H281	0.77154	0.79408	0.1582
C3	0.72402	0.10942	0.85292	C282	0.72112	0.62406	0.85121
C4	0.69329	0.19825	0.85617	C283	0.69033	0.71358	0.85388
C5	0.67818	0.29346	0.83415	C284	0.67551	0.80462	0.83139
C6	0.56748	0.27749	0.97846	C285	0.56438	0.786	0.97603
C7	0.53517	0.25531	0.97692	C286	0.53252	0.75427	0.97556
C8	0.52008	0.24464	0.95215	C287	0.51697	0.73334	0.95138
C9	0.58884	0.27637	0.95458	C288	0.58516	0.78704	0.95178
C10	0.43361	0.06352	0.92539	C289	0.42423	0.58016	0.92767
C11	0.41688	0.06287	0.90131	C290	0.40451	0.59857	0.90508
C12	0.38676	0.15956	0.89807	C291	0.37584	0.71128	0.9049
C13	0.37324	0.25445	0.91983	C292	0.36731	0.80814	0.92771
C14	0.39029	0.2574	0.94373	C293	0.38746	0.79312	0.94995
N15	0.64879	0.26945	0.88576	N294	0.64518	0.78571	0.88295
C16	0.67728	0.18828	0.88243	C295	0.67404	0.70783	0.88006
H17	0.53207	0.25246	0.9332	H296	0.52838	0.73872	0.93211
H18	0.45663	-0.01347	0.92714	H297	0.44549	0.48711	0.92756
H19	0.42742	-0.01403	0.88507	H298	0.41128	0.52185	0.88778
H20	0.34975	0.32763	0.91871	H299	0.34568	0.89859	0.92845
H21	0.37963	0.33569	0.95979	H300	0.38064	0.87181	0.96704
C22	0.30597	0.23569	0.78538	C301	0.27974	0.80093	0.80252
C23	0.29032	0.23185	0.75868	C302	0.77027	0.79557	0.27198
C24	0.25733	0.23073	0.75877	C303	0.73746	0.79506	0.27504
C25	0.33172	0.113	0.79388	C304	0.31134	0.72114	0.80549
C26	0.2917	0.35099	0.80432	C305	0.26472	0.88946	0.82419
C27	0.30522	0.23437	0.73303	C306	0.78187	0.80531	0.24517
H28	0.36554	0.02572	0.82515	H307	0.35141	0.65932	0.83148
H29	0.29231	0.44241	0.84428	H308	0.26871	0.96205	0.86508
H30	0.34123	0.01048	0.78109	H309	0.32348	0.6505	0.78925
H31	0.27075	0.44104	0.79904	H310	0.24068	0.95594	0.8222
H32	0.24469	0.22513	0.77764	H311	0.72732	0.78331	0.29491
N33	0.33774	0.23673	0.72556	N312	0.81345	0.80361	0.23485
H34	0.6898	0.11204	0.89865	H313	0.68668	0.63559	0.89657
C35	0.16878	0.33588	0.32553	C314	0.17109	0.82662	0.32863
C36	0.1842	0.24542	0.34782	C315	0.18666	0.73092	0.35041
C37	0.21514	0.15883	0.34476	C316	0.21744	0.64373	0.34679
C38	0.06288	0.30024	0.47331	C317	0.06522	0.79207	0.47565
C39	0.03109	0.26727	0.47402	C318	0.03302	0.7669	0.47557

C40	0.01415	0.24994	0.45041	C319	0.01636	0.7587	0.45152
C41	0.08236	0.30316	0.44828	C320	0.08519	0.793	0.45094
C42	0.92023	0.09517	0.42972	C321	0.92798	0.5802	0.42853
C43	0.89915	0.11775	0.40796	C322	0.90943	0.58685	0.40535
C44	0.87054	0.23064	0.40926	C323	0.87947	0.68697	0.40409
C45	0.86345	0.3224	0.43283	C324	0.86796	0.77793	0.42686
C46	0.88487	0.30278	0.45425	C325	0.88685	0.77412	0.4498
N47	0.13938	0.30975	0.37762	N326	0.14244	0.79524	0.38055
C48	0.16852	0.23749	0.37425	C327	0.17119	0.71871	0.37683
H49	0.02441	0.25939	0.43071	H328	0.02701	0.77049	0.43205
H50	0.94149	0.00272	0.4284	H329	0.95082	0.50066	0.42885
H51	0.90488	0.04458	0.39012	H330	0.91839	0.51288	0.38828
H52	0.84199	0.41282	0.43482	H331	0.84462	0.85284	0.42729
H53	0.87906	0.3779	0.47189	H332	0.87775	0.84945	0.46669
C54	0.76585	0.35065	0.31232	C333	0.78899	0.79481	0.29715
C55	0.7461	0.34808	0.28781	C334	0.26287	0.79083	0.77673
C56	0.71536	0.30543	0.28939	C335	0.23209	0.74842	0.77608
C57	0.75311	0.43103	0.33552	C336	0.77713	0.90799	0.31723
C58	0.79771	0.2719	0.31264	C337	0.8152	0.66953	0.30343
C59	0.75816	0.38697	0.26246	C338	0.27819	0.81799	0.75234
H60	0.76104	0.48957	0.3765	H339	0.7822	0.99277	0.35711
H61	0.84031	0.20359	0.33564	H340	0.85189	0.57484	0.33197
H62	0.72893	0.4968	0.33562	H341	0.75591	0.9998	0.3137
H63	0.80816	0.20765	0.29515	H342	0.82297	0.56872	0.28981
F64	0.70333	0.25914	0.31339	F343	0.217	0.71381	0.79924
F65	0.78694	0.43703	0.26035	F344	0.30714	0.86683	0.75221
H66	0.18203	0.16899	0.39066	H345	0.18442	0.6448	0.39291
C67	0.27825	0.34244	0.1467	C346	0.27869	0.83199	0.14865
C68	0.30887	0.25151	0.14367	C347	0.3095	0.74298	0.14594
C69	0.32363	0.1565	0.16586	C348	0.3245	0.65271	0.16842
C70	0.43548	0.16944	0.02182	C349	0.43544	0.65929	0.02399
C71	0.46759	0.1957	0.02337	C350	0.46735	0.68953	0.02442
C72	0.48243	0.21065	0.04811	C351	0.4829	0.70946	0.04859
C73	0.41398	0.16953	0.04563	C352	0.41463	0.66182	0.04823
C74	0.61277	0.19219	0.05673	C353	0.61271	0.67892	0.05162
C75	0.63016	0.20191	0.08029	C354	0.63185	0.67879	0.07433
C76	0.61739	0.30381	0.10132	C355	0.62131	0.77787	0.09627
C77	0.58687	0.3991	0.09806	C356	0.59126	0.88009	0.09492
C78	0.56981	0.39126	0.07435	C357	0.57261	0.88349	0.07192
N79	0.35355	0.17717	0.11425	N358	0.35453	0.67002	0.11696
C80	0.32511	0.25952	0.11746	C359	0.3257	0.74804	0.11974
H81	0.47037	0.20307	0.06703	H360	0.47154	0.70326	0.06787
H82	0.62316	0.10921	0.04114	H361	0.62129	0.59812	0.03518
H83	0.65362	0.12793	0.08231	H362	0.65489	0.59934	0.07498
H84	0.57609	0.48345	0.11322	H363	0.58206	0.96132	0.111
H85	0.54692	0.46866	0.07238	H364	0.55027	0.96718	0.07118
C86	0.68867	0.36353	0.21623	C365	0.70545	0.82763	0.20402
C87	0.70893	0.34995	0.24073	C366	0.23259	0.76364	0.72698

C88	0.74015	0.38502	0.23918	C367	0.26349	0.80289	0.72784
C89	0.65741	0.45023	0.21684	C368	0.67591	0.93299	0.20587
C90	0.70036	0.29156	0.19205	C369	0.7114	0.7229	0.18178
C91	0.69694	0.30674	0.26611	C370	0.21706	0.73518	0.7514
H92	0.61388	0.52549	0.1953	H371	0.63105	1.02332	0.18732
H93	0.69042	0.24051	0.15113	H372	0.69463	0.65169	0.14391
H94	0.64804	0.512	0.23489	H373	0.67034	1.01327	0.22297
H95	0.72435	0.22497	0.19099	H374	0.73338	0.63594	0.18016
F96	0.75301	0.42479	0.21526	F375	0.27935	0.83273	0.70495
F97	0.66761	0.26185	0.26833	F376	0.18779	0.68875	0.75141
H98	0.3128	0.3362	0.10119	H377	0.31303	0.81907	0.10312
C99	0.83168	0.12441	0.67366	C378	0.83025	0.62918	0.67181
C100	0.81614	0.21591	0.65151	C379	0.81488	0.7243	0.6499
C101	0.78521	0.30223	0.65473	C380	0.78407	0.81168	0.65331
C102	0.93726	0.15836	0.52599	C381	0.93705	0.66525	0.52498
C103	0.9691	0.19011	0.52526	C382	0.96913	0.69292	0.5251
C104	0.98606	0.20673	0.54886	C383	0.98565	0.70367	0.54915
C105	0.9178	0.15734	0.55103	C384	0.91698	0.66312	0.54965
C106	0.07844	0.37341	0.56941	C385	0.0743	0.88587	0.57162
C107	0.09946	0.36217	0.5911	C386	0.09305	0.88532	0.59454
C108	0.12908	0.25853	0.59014	C387	0.1233	0.78857	0.59589
C109	0.13717	0.16301	0.56708	C388	0.13452	0.69009	0.57381
C110	0.11577	0.17108	0.54572	C389	0.11544	0.68831	0.55108
N111	0.86084	0.15262	0.62167	N390	0.85935	0.65936	0.61989
C112	0.83172	0.22499	0.62507	C391	0.83055	0.73588	0.62352
H113	0.97585	0.19673	0.56856	H392	0.97495	0.69212	0.5686
H114	0.05642	0.45967	0.57046	H393	0.0514	0.9646	0.57113
H115	0.09298	0.43845	0.6085	H394	0.08413	0.96298	0.61123
H116	0.15966	0.08112	0.56521	H395	0.15749	0.61097	0.57403
H117	0.12246	0.09438	0.52839	H396	0.12435	0.60839	0.53463
C118	0.2355	0.21826	0.68514	C397	0.21661	0.75538	0.70074
C119	0.25485	0.23332	0.70957	C398	0.72852	0.82044	0.22644
C120	0.28773	0.24114	0.70942	C399	0.7614	0.82238	0.2233
C121	0.24649	0.10462	0.66487	C400	0.23274	0.67372	0.67906
C122	0.20464	0.31032	0.68288	C401	0.18344	0.83785	0.69649
C123	0.24012	0.22993	0.7349	C402	0.71718	0.80607	0.25289
H124	0.23709	0.00323	0.62683	H403	0.2308	0.60225	0.63808
H125	0.16238	0.36948	0.65922	H404	0.14471	0.88874	0.66886
H126	0.2697	0.0281	0.66623	H405	0.25694	0.61102	0.6814
H127	0.19521	0.3975	0.69827	H406	0.17039	0.90786	0.71234
N128	0.30886	0.2495	0.68713	N407	0.77974	0.83232	0.19925
H129	0.21515	0.22081	0.73645	H408	0.69238	0.79986	0.25658
H130	0.81822	0.29397	0.6087	H409	0.81741	0.80977	0.6074
C131	0.51745	0.24105	0.0009	C410	0.51586	0.73924	0.00014
C132	0.58096	0.29205	0.00371	C411	0.57851	0.80462	0.00084
H133	0.60534	0.31416	0.00462	H412	0.60261	0.83182	0.00117
C134	0.01596	0.24696	0.49921	C413	0.01707	0.74668	0.50034
C135	0.07841	0.31566	0.49761	C414	0.08022	0.80481	0.50027

H136	0.1025	0.34316	0.49703	H415	0.10452	0.8291	0.50016
C137	0.33791	0.24008	0.69828	C416	0.81015	0.8137	0.20771
H138	0.35915	0.24454	0.68645	H417	0.82991	0.81675	0.19406
C139	0.30197	0.30984	0.65932	C418	0.76984	0.90035	0.17236
C140	0.30396	0.35142	0.83027	C419	0.28077	0.89378	0.84857
C141	0.33136	0.2395	0.83786	C420	0.31201	0.80956	0.85167
C142	0.34506	0.11857	0.81951	C421	0.32727	0.72481	0.82979
C143	0.43884	0.17043	0.97251	C422	0.43652	0.66353	0.97476
C144	0.47082	0.20005	0.97393	C423	0.46869	0.68924	0.97494
C145	0.48861	0.22312	0.95069	C424	0.48583	0.70401	0.95107
C146	0.42104	0.16473	0.94669	C425	0.4163	0.67991	0.95016
C147	0.58097	0.38641	0.93203	C426	0.57606	0.88995	0.92866
C148	0.6008	0.38043	0.90963	C427	0.59608	0.8875	0.90633
C149	0.62971	0.27032	0.90964	C428	0.62605	0.78666	0.90684
C150	0.63844	0.16383	0.93259	C429	0.63579	0.68801	0.93034
C151	0.61809	0.16677	0.95475	C430	0.61552	0.68818	0.95243
N152	0.37089	0.15549	0.87263	N431	0.35663	0.72422	0.88108
C153	0.34492	0.25276	0.86488	C432	0.3284	0.81058	0.87768
H154	0.47876	0.22606	0.93077	H433	0.47562	0.69479	0.93154
H155	0.55933	0.47641	0.9316	H434	0.55338	0.97123	0.92769
H156	0.59383	0.46437	0.89226	H435	0.5883	0.9657	0.88855
H157	0.66032	0.07497	0.93345	H436	0.65849	0.60672	0.93179
H158	0.62495	0.08075	0.9719	H437	0.62333	0.60733	0.96995
C159	0.72409	0.20737	0.80595	C438	0.72183	0.71302	0.80388
C160	0.7399	0.20839	0.77934	C439	0.73808	0.70991	0.77742
C161	0.77104	0.24438	0.77702	C440	0.76934	0.74433	0.77528
C162	0.69345	0.29786	0.80929	C441	0.69113	0.80427	0.80665
C163	0.73932	0.11423	0.82807	C442	0.73674	0.62391	0.82647
C164	0.72393	0.17291	0.75601	C443	0.72234	0.67348	0.75402
H165	0.65455	0.36449	0.83607	H444	0.65185	0.87598	0.83288
H166	0.7361	0.0347	0.86957	H445	0.73298	0.55232	0.86823
H167	0.6814	0.37309	0.7927	H446	0.67927	0.87625	0.78968
H168	0.76286	0.0427	0.82602	H447	0.76027	0.55152	0.82487
F169	0.78692	0.28284	0.79901	F448	0.78512	0.78277	0.79732
F170	0.69419	0.13314	0.75757	F449	0.6926	0.63338	0.75545
H171	0.33317	0.35075	0.87763	H450	0.3162	0.88201	0.89374
C172	0.81606	0.2682	0.33591	C451	0.83117	0.66982	0.32799
C173	0.80296	0.34413	0.35935	C452	0.81975	0.78823	0.34749
C174	0.7715	0.42779	0.3588	C453	0.79207	0.90353	0.34214
C175	0.93542	0.17039	0.4767	C454	0.93704	0.67571	0.47563
C176	0.96747	0.19839	0.47572	C455	0.96892	0.70677	0.47557
C177	0.98312	0.21856	0.45124	C456	0.98498	0.73472	0.45153
C178	0.9137	0.19031	0.45292	C457	0.91738	0.67762	0.45078
C179	0.07224	0.40854	0.42564	C458	0.07611	0.90361	0.42868
C180	0.09121	0.40815	0.40263	C459	0.095	0.90035	0.40569
C181	0.12114	0.30717	0.40201	C460	0.12413	0.79246	0.40483
C182	0.13179	0.20501	0.42495	C461	0.13393	0.68471	0.42741
C183	0.11257	0.20311	0.4477	C462	0.11456	0.68492	0.45013

N184	0.8499	0.24946	0.38617	N463	0.86156	0.69012	0.37959
C185	0.82152	0.3369	0.38447	C464	0.83589	0.79502	0.37349
H186	0.97181	0.21149	0.4321	H465	0.97381	0.73921	0.43212
H187	0.04965	0.49072	0.42561	H466	0.05434	0.99249	0.42896
H188	0.0827	0.48873	0.38528	H467	0.0872	0.98512	0.38861
H189	0.15437	0.12231	0.42546	H468	0.15593	0.5968	0.4276
H190	0.1211	0.11989	0.46478	H469	0.12231	0.59813	0.467
C191	0.21493	0.24985	0.29747	C470	0.21672	0.74436	0.29995
C192	0.23096	0.2493	0.27089	C471	0.23226	0.74763	0.27327
C193	0.26223	0.28369	0.26871	C472	0.26334	0.78429	0.27091
C194	0.23036	0.16106	0.31987	C473	0.23235	0.65056	0.32182
C195	0.18404	0.33812	0.30061	C474	0.18601	0.83324	0.30364
C196	0.21517	0.21327	0.24747	C475	0.21626	0.71186	0.24997
H197	0.22746	0.08759	0.36161	H476	0.22985	0.56866	0.36325
H198	0.14499	0.40508	0.32733	H477	0.14741	0.89646	0.33087
H199	0.25404	0.09095	0.31805	H478	0.25594	0.5802	0.3195
H200	0.17177	0.40995	0.28381	H479	0.17367	0.90896	0.28722
F201	0.27806	0.32211	0.29072	F480	0.27929	0.82253	0.29287
F202	0.18545	0.17299	0.24891	F481	0.18658	0.67127	0.25159
H203	0.81054	0.40334	0.4015	H482	0.82624	0.89406	0.38695
C204	0.6809	0.30018	0.16929	C483	0.68912	0.73138	0.16108
C205	0.64932	0.38222	0.17029	C484	0.66	0.84053	0.16275
C206	0.63796	0.45909	0.19415	C485	0.65343	0.93906	0.1855
C207	0.56417	0.27843	0.02776	C486	0.56312	0.7838	0.02531
C208	0.53189	0.25322	0.02636	C487	0.5311	0.75535	0.02507
C209	0.51371	0.23584	0.04957	C488	0.51396	0.7392	0.04892
C210	0.58207	0.28547	0.05353	C489	0.58264	0.78063	0.05021
C211	0.42206	0.0622	0.06845	C490	0.42332	0.55811	0.07133
C212	0.40201	0.0678	0.09074	C491	0.4033	0.56321	0.09365
C213	0.37277	0.17532	0.09041	C492	0.3737	0.66749	0.09312
C214	0.36387	0.27859	0.06716	C493	0.3644	0.76801	0.06971
C215	0.38434	0.27568	0.04509	C494	0.38472	0.76528	0.04762
N216	0.63638	0.30897	0.12511	N495	0.64204	0.77251	0.11908
C217	0.62766	0.38835	0.14703	C496	0.63621	0.85492	0.14112
H218	0.52325	0.2379	0.06953	H497	0.52406	0.74687	0.06851
H219	0.44406	-0.02487	0.06923	H498	0.44567	0.47403	0.07228
H220	0.40913	-0.01387	0.10832	H499	0.41075	0.48403	0.11141
H221	0.34171	0.36502	0.06594	H500	0.34204	0.85256	0.06837
H222	0.37729	0.35949	0.02773	H501	0.37729	0.84716	0.03012
C223	0.27758	0.24668	0.19376	C502	0.27831	0.74405	0.19603
C224	0.26148	0.24749	0.22028	C503	0.26226	0.7474	0.22254
C225	0.23021	0.21324	0.22242	C504	0.2311	0.71173	0.22486
C226	0.30812	0.15424	0.19064	C505	0.30902	0.65329	0.19321
C227	0.26273	0.33985	0.17148	C506	0.26321	0.83233	0.17345
C228	0.2773	0.28278	0.2437	C507	0.27813	0.78494	0.2458
H229	0.34716	0.08399	0.16411	H508	0.34818	0.58169	0.1669
H230	0.26645	0.41721	0.12994	H509	0.26671	0.90302	0.13162
H231	0.3199	0.07915	0.20736	H510	0.32101	0.58188	0.21017

H232	0.23931	0.41323	0.17336	H511	0.23963	0.90406	0.17509
F233	0.21441	0.17547	0.20035	F512	0.21529	0.67158	0.20295
F234	0.30716	0.32101	0.2423	F513	0.30779	0.82594	0.24418
H235	0.60404	0.45807	0.14912	H514	0.61405	0.93813	0.14407
C236	0.18583	0.2947	0.66047	C515	0.16904	0.82818	0.67161
C237	0.19738	0.18535	0.63984	C516	0.18593	0.74254	0.65046
C238	0.2278	0.08999	0.64233	C517	0.21766	0.66814	0.6543
C239	0.06464	0.28874	0.52264	C518	0.06522	0.784	0.52498
C240	0.03268	0.25915	0.52358	C519	0.03319	0.75502	0.5251
C241	0.01705	0.23818	0.54805	C520	0.01691	0.73036	0.54914
C242	0.08605	0.27607	0.54659	C521	0.08487	0.78474	0.54979
C243	0.92774	0.05125	0.57363	C522	0.92623	0.55403	0.57207
C244	0.90883	0.05266	0.59665	C523	0.90716	0.55636	0.59497
C245	0.87909	0.15533	0.59732	C524	0.87777	0.66218	0.59564
C246	0.86864	0.259	0.57448	C525	0.86783	0.76813	0.5729
C247	0.88785	0.26	0.55171	C526	0.88731	0.76857	0.55026
N248	0.15016	0.25822	0.61265	N527	0.14134	0.79344	0.6201
C249	0.17823	0.16911	0.61568	C528	0.17082	0.72843	0.62415
H250	0.02831	0.24464	0.5672	H529	0.02789	0.72933	0.5686
H251	0.95014	-0.03247	0.5736	H530	0.94828	0.46735	0.572
H252	0.91718	-0.02879	0.61395	H531	0.91509	0.47286	0.61216
H253	0.84622	0.34326	0.57408	H532	0.84562	0.85426	0.57251
H254	0.8795	0.34402	0.53467	H533	0.87941	0.85422	0.53329
C255	0.78572	0.2093	0.70193	C534	0.78434	0.71224	0.70021
C256	0.76992	0.20901	0.72858	C535	0.76852	0.70952	0.72682
C257	0.73876	0.17332	0.73091	C536	0.73733	0.67398	0.72898
C258	0.77014	0.29889	0.67967	C537	0.76893	0.80555	0.67819
C259	0.81656	0.12106	0.69862	C538	0.81509	0.62325	0.69673
C260	0.78584	0.24533	0.7519	C539	0.78437	0.74445	0.75025
H261	0.77281	0.37413	0.63798	H540	0.7718	0.88638	0.63675
H262	0.85545	0.05529	0.67173	H541	0.85395	0.55926	0.66974
H263	0.74646	0.36875	0.68165	H542	0.74532	0.87612	0.68033
H264	0.82891	0.04844	0.71532	H543	0.8273	0.54793	0.71324
F265	0.72282	0.13476	0.70897	F544	0.72149	0.63658	0.70691
F266	0.81548	0.28665	0.75029	F545	0.81411	0.78443	0.74882
H267	0.18832	0.08185	0.60061	H546	0.18495	0.66383	0.60842
C268	0.48531	0.21092	-0.00059	C547	0.48403	0.70424	-0.00016
C269	0.42221	0.15282	0.99653	C548	0.42121	0.6415	0.99923
H270	0.39802	0.12752	0.99557	H549	0.39691	0.61734	0.99892
C271	0.98424	0.21008	0.50006	C550	0.98508	0.71392	0.50034
C272	0.92165	0.14348	0.50173	C551	0.92216	0.65189	0.50031
H273	0.89742	0.1181	0.50232	H552	0.89798	0.62586	0.50035
H274	0.31691	0.40814	0.65336	H553	0.78546	0.9922	0.16502
C275	0.36645	0.20571	0.74199	C554	0.844	0.76384	0.24857
H276	0.38696	0.23861	0.73021	H555	0.86325	0.79519	0.23508
H277	0.37207	0.06794	0.74787	H556	0.84929	0.62481	0.25362
H278	0.36374	0.28748	0.75976	H557	0.84416	0.84172	0.26665
I279	0.351	0.61622	0.71854	I558	0.82789	1.17942	0.22687

Supplementary Table 2. Fractional atomic coordinates in the refined unit cell of Py-TFIm-25 COF.

Py-TFIm-25 COF: Space group: P1							
a = 44.98 Å, b = 7.93 Å, c = 47.37 Å							
α = 88.52°, β = 90.90°, and γ = 85.17°							
Atom	x	y	z	Atom	x	y	z
H1	0.26392	0.22533	0.67173	H275	0.76632	0.97148	0.16448
H2	0.28491	0.02675	0.66627	H276	0.79403	0.80429	0.15693
C3	0.67078	0.14401	0.82605	C277	0.66933	0.64382	0.8269
C4	0.6816	0.21179	0.85058	C278	0.68015	0.7125	0.85136
C5	0.71073	0.26219	0.85044	C279	0.70921	0.76394	0.85111
C6	0.56201	0.28993	0.97242	C280	0.561	0.79028	0.97351
C7	0.53035	0.28767	0.97292	C281	0.52935	0.78857	0.97426
C8	0.51359	0.25878	0.94861	C282	0.51239	0.76051	0.95005
C9	0.5804	0.27013	0.94709	C283	0.57923	0.77045	0.94808
C10	0.42472	0.12382	0.92999	C284	0.42315	0.63177	0.93162
C11	0.4065	0.10376	0.90724	C285	0.4048	0.61424	0.90886
C12	0.37836	0.19369	0.90508	C286	0.37679	0.70565	0.90697
C13	0.36919	0.30464	0.92662	C287	0.36789	0.81537	0.92875
C14	0.38782	0.32664	0.949	C288	0.38668	0.83494	0.95111
N15	0.63512	0.21685	0.87544	N289	0.63373	0.71714	0.87629
C16	0.66335	0.2348	0.87566	C290	0.66194	0.7355	0.87646
H17	0.52518	0.23697	0.92885	H291	0.52382	0.73849	0.93021
H18	0.44562	0.04392	0.93177	H292	0.44389	0.55032	0.9332
H19	0.41361	0.0126	0.89114	H293	0.41171	0.52381	0.8926
H20	0.34753	0.37765	0.92529	H294	0.3463	0.88907	0.92764
H21	0.38046	0.41825	0.96498	H295	0.37952	0.92582	0.96727
C22	0.28351	0.16107	0.80472	C296	0.27883	0.67638	0.80864
C23	0.26713	0.15414	0.77838	C297	0.77404	0.78838	0.26863
C24	0.23589	0.17062	0.77688	C298	0.74347	0.76718	0.2665
C25	0.27049	0.12441	0.83046	C299	0.26906	0.60583	0.83361
C26	0.3131	0.20609	0.80496	C300	0.3065	0.74619	0.80862
C27	0.28162	0.13612	0.75238	C301	0.78795	0.82731	0.24326
H28	0.27602	0.09608	0.87473	H302	0.27839	0.54772	0.87668
H29	0.35209	0.24398	0.82925	H303	0.34536	0.79849	0.83208
H30	0.24759	0.08809	0.8312	H304	0.24779	0.54876	0.83433
H31	0.32356	0.23903	0.78535	H305	0.3144	0.806	0.78973
H32	0.22293	0.18634	0.79603	H306	0.73109	0.73613	0.28519
N33	0.31196	0.1175	0.74749	N307	0.8169	0.8696	0.23951
H34	0.67493	0.27756	0.8946	H308	0.67353	0.77876	0.89536
C35	0.16747	0.3807	0.32645	C309	0.16704	0.87695	0.32881
C36	0.17756	0.26848	0.34795	C310	0.17695	0.76352	0.35021
C37	0.20516	0.17563	0.34443	C311	0.20448	0.67004	0.34665
C38	0.06374	0.24715	0.4744	C312	0.06195	0.74875	0.4759
C39	0.03228	0.23552	0.47403	C313	0.03052	0.73562	0.47518
C40	0.01593	0.23332	0.44874	C314	0.01452	0.73132	0.44972
C41	0.08201	0.26867	0.44935	C315	0.08054	0.76863	0.45101
C42	0.92805	0.09319	0.42423	C316	0.92691	0.5924	0.42462

C43	0.90997	0.09288	0.40091	C317	0.90934	0.59223	0.40096
C44	0.88246	0.19048	0.39954	C318	0.88206	0.6917	0.39912
C45	0.87371	0.28762	0.42295	C319	0.87303	0.79134	0.42216
C46	0.89175	0.28596	0.44633	C320	0.89061	0.78987	0.44584
N47	0.13595	0.33847	0.37812	N321	0.13531	0.83493	0.38024
C48	0.16005	0.24262	0.37296	C322	0.15923	0.73749	0.3751
H49	0.02768	0.24024	0.42893	H323	0.02653	0.73656	0.43003
H50	0.94874	0.01066	0.4248	H324	0.9474	0.50835	0.42571
H51	0.91695	0.0134	0.38323	H325	0.91646	0.51108	0.38353
H52	0.85268	0.367	0.42291	H326	0.85223	0.8727	0.42138
H53	0.88448	0.36437	0.46409	H327	0.88325	0.87022	0.46334
C54	0.78855	0.27126	0.29682	C328	0.79026	0.77244	0.29514
C55	0.7709	0.28455	0.27068	C329	0.26058	0.67685	0.78293
C56	0.74051	0.25673	0.26971	C330	0.22927	0.69151	0.78294
C57	0.7761	0.33323	0.32181	C331	0.77573	0.80367	0.32068
C58	0.81795	0.19825	0.29728	C332	0.82102	0.72521	0.2958
C59	0.78301	0.32849	0.24489	C333	0.27324	0.66254	0.75621
H60	0.78253	0.37561	0.36537	H334	0.7796	0.81917	0.3651
H61	0.85712	0.12936	0.32188	H335	0.86037	0.67273	0.32085
H62	0.75333	0.39218	0.32201	H336	0.75193	0.84319	0.32093
H63	0.82791	0.14547	0.27823	H337	0.83271	0.6977	0.27627
F64	0.7265	0.2087	0.29324	F338	0.21419	0.7094	0.80739
F65	0.81209	0.3615	0.24329	F339	0.30327	0.64225	0.7532
H66	0.16849	0.13501	0.38678	H340	0.16725	0.62837	0.38879
C67	0.32742	0.42253	0.1729	C341	0.32728	0.92035	0.17541
C68	0.31734	0.35762	0.14784	C342	0.31703	0.85809	0.15021
C69	0.28889	0.29753	0.14747	C343	0.28843	0.80001	0.14976
C70	0.43578	0.33081	0.02553	C344	0.43517	0.83274	0.02748
C71	0.46728	0.33988	0.02463	C345	0.46671	0.84037	0.02643
C72	0.48359	0.39067	0.04802	C346	0.48328	0.88898	0.0498
C73	0.41772	0.34738	0.05103	C347	0.41724	0.84876	0.05308
C74	0.57369	0.27349	0.07358	C348	0.57448	0.77584	0.07468
C75	0.59199	0.28005	0.09662	C349	0.59361	0.78181	0.09716
C76	0.61879	0.359	0.0951	C350	0.6206	0.85795	0.09464
C77	0.62621	0.43543	0.06978	C351	0.62725	0.93345	0.06904
C78	0.60762	0.42974	0.04686	C352	0.60779	0.92883	0.04675
N79	0.36346	0.378	0.12318	N353	0.36317	0.87686	0.12543
C80	0.33578	0.34611	0.12279	C354	0.33538	0.8473	0.12508
H81	0.47152	0.43313	0.06674	H355	0.4714	0.92996	0.0687
H82	0.55379	0.20324	0.07506	H356	0.5545	0.70691	0.07689
H83	0.58612	0.21801	0.11608	H357	0.58825	0.72082	0.11685
H84	0.64653	0.50203	0.0682	H358	0.64771	0.99797	0.06676
H85	0.61367	0.49263	0.02748	H359	0.61332	0.99108	0.02716
C86	0.71761	0.33247	0.19346	C360	0.7233	0.82131	0.18998
C87	0.73567	0.31767	0.21937	C361	0.22487	0.67858	0.73185
C88	0.7661	0.34533	0.22041	C362	0.25617	0.66306	0.73188
C89	0.73025	0.29014	0.16756	C363	0.73258	0.73504	0.16574
C90	0.68721	0.38634	0.19409	C364	0.69487	0.90789	0.18989

C91	0.72372	0.2713	0.24516	C365	0.21221	0.69291	0.75855
H92	0.72345	0.25978	0.12369	H366	0.72193	0.6664	0.12397
H93	0.64635	0.4341	0.17105	H367	0.65454	0.97797	0.16703
H94	0.7539	0.24854	0.16617	H368	0.75407	0.66021	0.16547
H95	0.67674	0.42498	0.21375	H369	0.68711	0.97702	0.20845
F96	0.78025	0.39467	0.19689	F370	0.27129	0.65259	0.70726
F97	0.69481	0.23372	0.24676	F371	0.1821	0.7043	0.76147
H98	0.32504	0.29914	0.1037	H372	0.32441	0.80291	0.10593
C99	0.8321	0.09194	0.67251	C373	0.82974	0.60142	0.67259
C100	0.82313	0.20201	0.65036	C374	0.82024	0.70898	0.65029
C101	0.79616	0.30226	0.65313	C375	0.79309	0.80743	0.65315
C102	0.93769	0.18096	0.52431	C376	0.93532	0.68346	0.52452
C103	0.96918	0.19209	0.52472	C377	0.96685	0.6922	0.52522
C104	0.98552	0.19564	0.55	C378	0.98294	0.69272	0.55067
C105	0.91917	0.16458	0.54934	C379	0.9166	0.6686	0.54945
C106	0.07443	0.32232	0.5747	C380	0.06945	0.83402	0.57626
C107	0.09322	0.32275	0.59754	C381	0.08719	0.83856	0.59974
C108	0.12081	0.22603	0.59814	C382	0.11533	0.74819	0.60123
C109	0.12925	0.13277	0.57427	C383	0.12545	0.65766	0.57758
C110	0.11045	0.1338	0.55142	C384	0.10764	0.65397	0.55412
N111	0.86431	0.11459	0.62053	N385	0.86158	0.62349	0.62064
C112	0.84125	0.2193	0.62547	C386	0.83793	0.72466	0.62511
H113	0.97374	0.19182	0.56982	H387	0.971	0.68528	0.57036
H114	0.05375	0.40494	0.57471	H388	0.04825	0.91185	0.57554
H115	0.08659	0.40058	0.61551	H389	0.07929	0.91458	0.61753
H116	0.1508	0.05888	0.57339	H390	0.1475	0.58903	0.57736
H117	0.11745	0.05768	0.53332	H391	0.1159	0.57954	0.53625
C118	0.21585	0.14748	0.70021	C392	0.2069	0.67769	0.70598
C119	0.23426	0.15313	0.7256	C393	0.7416	0.81441	0.21562
C120	0.26566	0.13863	0.7268	C394	0.77222	0.8382	0.21755
C121	0.22319	0.0331	0.67863	C395	0.217	0.57984	0.68318
C122	0.1893	0.25229	0.69746	C396	0.17933	0.77275	0.70348
C123	0.22037	0.17103	0.75174	C397	0.7281	0.78058	0.24126
H124	0.21153	-0.0641	0.63889	H398	0.20825	0.49566	0.64186
H125	0.15116	0.33226	0.67216	H399	0.14129	0.84602	0.67766
H126	0.24274	-0.05759	0.68074	H400	0.23814	0.50206	0.6845
H127	0.1827	0.34149	0.71402	H401	0.17098	0.85207	0.72069
N128	0.2876	0.12837	0.70656	N402	0.79288	0.88796	0.19839
H129	0.19605	0.18305	0.75242	H403	0.7044	0.75994	0.24126
H130	0.83435	0.32941	0.61156	H404	0.83004	0.82945	0.61051
C131	0.51463	0.31374	0.99844	C405	0.51388	0.8143	0.99992
C132	0.57687	0.31061	-0.00244	C406	0.57608	0.81067	-0.00147
H133	0.60118	0.30727	-0.00273	H407	0.60039	0.80714	-0.002
C134	0.01653	0.22024	0.49955	C408	0.01444	0.72105	0.50053
C135	0.07841	0.23772	0.50012	C409	0.0762	0.74369	0.50181
H136	0.10245	0.24901	0.50039	H410	0.10014	0.75779	0.50236
C137	0.31452	0.11555	0.71997	C411	0.81891	0.90424	0.21247
H138	0.33558	0.10497	0.70867	H412	0.83877	0.94418	0.20208

C139	0.28468	0.14998	0.67683	C413	0.78916	0.92102	0.16901
C140	0.32909	0.20927	0.82948	C414	0.32387	0.74336	0.83246
C141	0.31632	0.16861	0.85504	C415	0.31428	0.67164	0.85736
C142	0.28655	0.12797	0.85503	C416	0.28642	0.60441	0.85747
C143	0.43527	0.27189	0.97525	C417	0.43423	0.7764	0.97711
C144	0.46689	0.27758	0.97433	C418	0.46588	0.78055	0.97606
C145	0.48326	0.25782	0.94919	C419	0.48205	0.76074	0.95081
C146	0.41633	0.24013	0.9511	C420	0.41509	0.74703	0.95297
C147	0.57269	0.35499	0.92187	C421	0.57139	0.85482	0.92285
C148	0.59095	0.33796	0.8988	C422	0.58956	0.83769	0.89972
C149	0.61825	0.23882	0.90007	C423	0.61693	0.73917	0.90094
C150	0.6263	0.15441	0.9252	C424	0.62513	0.65536	0.92608
C151	0.60778	0.1711	0.94813	C425	0.60667	0.67193	0.94906
N152	0.36191	0.18316	0.8803	N426	0.36	0.69967	0.88226
C153	0.33357	0.16807	0.88085	C427	0.33243	0.66699	0.88274
H154	0.47141	0.24206	0.92964	H428	0.47005	0.74573	0.93131
H155	0.55238	0.44044	0.92059	H429	0.55104	0.93995	0.92159
H156	0.5846	0.40703	0.87957	H430	0.58309	0.90624	0.88047
H157	0.64712	0.07338	0.92657	H431	0.646	0.57483	0.92743
H158	0.61436	0.10205	0.96735	H432	0.61336	0.60319	0.96829
C159	0.71691	0.18623	0.80194	C433	0.71527	0.68788	0.80261
C160	0.73494	0.18076	0.77613	C434	0.73314	0.68355	0.7767
C161	0.76617	0.15289	0.77604	C435	0.76438	0.65753	0.77645
C162	0.72805	0.25032	0.82666	C436	0.72644	0.75254	0.82726
C163	0.68806	0.13129	0.80224	C437	0.68652	0.63154	0.80304
C164	0.7222	0.20808	0.74951	C438	0.72019	0.71059	0.75016
H165	0.71979	0.3149	0.86936	H439	0.71828	0.81722	0.86998
H166	0.64831	0.1013	0.82583	H440	0.64693	0.60014	0.82679
H167	0.75034	0.29504	0.82715	H441	0.74867	0.79818	0.82765
H168	0.67902	0.07643	0.78358	H442	0.6775	0.576	0.78443
F169	0.78117	0.12005	0.80035	F443	0.77958	0.62499	0.80066
F170	0.69228	0.24133	0.74676	F444	0.69024	0.74212	0.74758
H171	0.32142	0.14771	0.90067	H445	0.3214	0.62785	0.90232
C172	0.83433	0.18832	0.32178	C446	0.83652	0.71111	0.32069
C173	0.82212	0.25208	0.34678	C447	0.8221	0.74458	0.34616
C174	0.79261	0.32421	0.3462	C448	0.79134	0.79102	0.34554
C175	0.93789	0.19473	0.47306	C449	0.93611	0.69654	0.47332
C176	0.96937	0.20573	0.47335	C450	0.96761	0.70582	0.47388
C177	0.9858	0.22238	0.44843	C451	0.98438	0.72072	0.44911
C178	0.91963	0.19204	0.4475	C452	0.91823	0.69361	0.44756
C179	0.073	0.37876	0.42715	C453	0.07168	0.87632	0.42846
C180	0.09123	0.39775	0.40438	C454	0.09017	0.89409	0.40585
C181	0.11938	0.30787	0.40245	C455	0.11844	0.80516	0.4044
C182	0.1289	0.20161	0.425	C456	0.12783	0.70149	0.4273
C183	0.11062	0.18357	0.44775	C457	0.10928	0.68474	0.44991
N184	0.86666	0.18788	0.37406	N458	0.86671	0.69319	0.37346
C185	0.83888	0.24552	0.37289	C459	0.83821	0.7323	0.37258
H186	0.9741	0.22685	0.42844	H460	0.97294	0.72409	0.42899

H187	0.05185	0.45666	0.42828	H461	0.05044	0.95357	0.42922
H188	0.08377	0.48545	0.38753	H462	0.08282	0.98006	0.38873
H189	0.15081	0.13139	0.4249	H463	0.14986	0.63254	0.42758
H190	0.11842	0.09702	0.46468	H464	0.11699	0.60028	0.46714
C191	0.21158	0.30442	0.29898	C465	0.21115	0.80042	0.30135
C192	0.22925	0.32101	0.27323	C466	0.22888	0.81769	0.27565
C193	0.26058	0.30292	0.27295	C467	0.2602	0.79884	0.27535
C194	0.22193	0.19383	0.32062	C468	0.22134	0.68872	0.32291
C195	0.18399	0.39748	0.30244	C469	0.18364	0.89423	0.30486
C196	0.2162	0.35475	0.24686	C470	0.21587	0.85321	0.24931
H197	0.21359	0.08657	0.36079	H471	0.21279	0.58009	0.36294
H198	0.14608	0.45472	0.32876	H472	0.14571	0.95156	0.33115
H199	0.24309	0.11759	0.31859	H473	0.24244	0.61196	0.32085
H200	0.17547	0.48676	0.28624	H474	0.17523	0.98446	0.28875
F201	0.27604	0.27386	0.29711	F475	0.27564	0.76812	0.29948
F202	0.18611	0.37121	0.24412	F476	0.18579	0.87048	0.24653
H203	0.82615	0.29175	0.39159	H477	0.82458	0.75513	0.39174
C204	0.67006	0.39273	0.17007	C478	0.67659	0.9094	0.16664
C205	0.68247	0.34538	0.14432	C479	0.68584	0.82309	0.14244
C206	0.71303	0.29628	0.14356	C480	0.71427	0.73685	0.14248
C207	0.56233	0.33682	0.023	C481	0.56184	0.83687	0.02412
C208	0.53058	0.34459	0.02331	C482	0.53008	0.84427	0.02471
C209	0.51391	0.39241	0.04743	C483	0.5136	0.89058	0.049
C210	0.58085	0.34956	0.0481	C484	0.5809	0.85025	0.04886
C211	0.4275	0.27582	0.07673	C485	0.42694	0.77452	0.07862
C212	0.40945	0.28625	0.09999	C486	0.40894	0.78403	0.10192
C213	0.38038	0.36481	0.09849	C487	0.38	0.8646	0.10066
C214	0.3704	0.43741	0.07296	C488	0.37012	0.94014	0.07532
C215	0.38869	0.42765	0.04985	C489	0.38835	0.93103	0.05214
N216	0.6357	0.36717	0.11984	N490	0.63865	0.86813	0.11862
C217	0.66433	0.34074	0.11901	C491	0.66648	0.81574	0.11795
H218	0.52553	0.43589	0.06571	H492	0.52538	0.93267	0.06729
H219	0.44942	0.20597	0.07826	H493	0.44874	0.70326	0.07996
H220	0.41744	0.22747	0.11961	H494	0.41688	0.72302	0.12139
H221	0.34815	0.50327	0.07139	H495	0.348	1.00773	0.07392
H222	0.38049	0.48695	0.03029	H496	0.38019	0.99253	0.03273
C223	0.28207	0.35792	0.19648	C497	0.28178	0.85707	0.19892
C224	0.2642	0.34941	0.22226	C498	0.2639	0.84785	0.22471
C225	0.2329	0.36778	0.22251	C499	0.2326	0.86715	0.22499
C226	0.27157	0.29735	0.17124	C500	0.27113	0.79912	0.17355
C227	0.31017	0.42272	0.19672	C501	0.31004	0.91995	0.19925
C228	0.27726	0.31669	0.24863	C502	0.27691	0.81341	0.25105
H229	0.2804	0.24705	0.12812	H503	0.27978	0.75162	0.13032
H230	0.34933	0.47262	0.17352	H504	0.3493	0.96897	0.17614
H231	0.24985	0.2455	0.17034	H505	0.24929	0.74883	0.17253
H232	0.31862	0.4752	0.21584	H506	0.31865	0.97048	0.21846
F233	0.21759	0.40436	0.19848	F507	0.21729	0.90549	0.201
F234	0.30732	0.2914	0.25111	F508	0.30696	0.78717	0.25355

H235	0.67647	0.30441	0.09946	H509	0.67689	0.75426	0.09914
C236	0.17159	0.24819	0.6739	C510	0.16265	0.77046	0.67923
C237	0.17924	0.13642	0.6521	C511	0.17249	0.67061	0.65657
C238	0.20528	0.02766	0.65522	C512	0.20002	0.57533	0.65916
C239	0.06381	0.22701	0.52563	C513	0.06121	0.73378	0.52713
C240	0.03227	0.21841	0.5254	C514	0.0298	0.7206	0.52654
C241	0.01573	0.20374	0.5503	C515	0.01311	0.70195	0.55126
C242	0.08239	0.22603	0.55104	C516	0.079	0.73905	0.55289
C243	0.92788	0.05995	0.57231	C517	0.92461	0.55966	0.57207
C244	0.90942	0.04712	0.59508	C518	0.906	0.54843	0.59477
C245	0.88131	0.13806	0.59623	C519	0.87848	0.64541	0.5962
C246	0.87197	0.23759	0.57285	C520	0.8699	0.7503	0.57321
C247	0.89052	0.24981	0.55015	C521	0.88853	0.76038	0.55052
N248	0.1385	0.23955	0.62238	N522	0.13168	0.76388	0.62622
C249	0.16048	0.1267	0.62733	C523	0.15484	0.65934	0.63117
H250	0.02736	0.1994	0.57032	H524	0.02461	0.69465	0.57135
H251	0.94897	-0.01883	0.57182	H525	0.94522	0.47666	0.57135
H252	0.91669	-0.03612	0.61256	H526	0.91268	0.46162	0.61197
H253	0.84998	0.30695	0.57229	H527	0.84847	0.82572	0.5729
H254	0.88288	0.33173	0.53259	H528	0.88151	0.84588	0.5332
C255	0.7883	0.186	0.69923	C529	0.78597	0.69312	0.69952
C256	0.77034	0.18156	0.72502	C530	0.7682	0.68759	0.72541
C257	0.73909	0.2087	0.72512	C531	0.73692	0.7127	0.72568
C258	0.77896	0.29358	0.67687	C532	0.7762	0.79909	0.67709
C259	0.8152	0.08506	0.69649	C533	0.8131	0.59439	0.69672
C260	0.78308	0.15286	0.75164	C534	0.78113	0.65909	0.75196
H261	0.78858	0.38939	0.6362	H535	0.78514	0.89303	0.63615
H262	0.85297	0.01213	0.67079	H536	0.85078	0.52323	0.67082
H263	0.75832	0.37533	0.6783	H537	0.75542	0.87952	0.67859
H264	0.82289	-0.0023	0.71326	H538	0.82116	0.5085	0.71356
F265	0.72389	0.23386	0.70074	F539	0.72153	0.73739	0.7014
F266	0.8131	0.12846	0.75454	F540	0.81119	0.63651	0.75472
H267	0.16601	0.01598	0.61357	H541	0.16241	0.5547	0.61683
C268	0.48291	0.30948	0.99915	C542	0.48213	0.81079	1.00082
C269	0.42097	0.29937	0.00077	C543	0.42014	0.80346	0.00275
H270	0.39696	0.28803	0.00171	H544	0.3961	0.79349	0.00379
C271	0.98502	0.20544	0.49921	C545	0.98297	0.70575	0.49988
C272	0.92317	0.18641	0.49852	C546	0.92103	0.69042	0.49863
H273	0.89916	0.17427	0.49817	H547	0.89695	0.68074	0.49808
H274	0.30347	0.21635	0.66899	H548	0.80459	1.01391	0.16238

Supplementary Table 3. Pore parameters of Py-TFImI-25 COF and Py-TFIm-25 COF.

Adsorbents	Surface area (m^2/g)	Microporous pore volume (cm^3/g)	Pore volume (cm^3/g)
Py-TFImI-25	1324.05	0.013	0.68
Py-TFIm-25	1430.15	0.028	0.74

Supplementary Table 4. Th(IV) adsorption performances over various materials.

Category	Sorbents	Equilibrium time	c_0 (mg L^{-1})	q_e (mg g^{-1})	Ref.
Zeolite	NKF-6	3 h	40	51	17
Polymer	P (DVB-VPA)	1 h	26	54	18
Porous silica	SBA/SA	5 h	50	12	19
	SBA/EnSA	2 h	50	49	
GO	Reduced graphene oxide	50 min	35	12	20
MOF	MOFs with O^- groups	10 min	41	67	21
Composites	Tannin-modified poly(glycidylmethacrylate)-grafted zirconium oxide-densified cellulose (TMPGZDC)	2 h	50	23	22
	Fe_3O_4@AMCA-MIL53(Al)	90 min	20	22	23
**COF**	$[NH_4]^+[COF\text{-}SO_3^-]$	1 h	50	100	24
	COF-DL229	1 min	50	133	25
	**Py-TFImI-25 COF**	**4 min**	**50**	**136**	**This work**
	**Py-TFIm-25 COF**	**1 min**	**47**	**141**	

Supplementary Table 5. Analysis of nitrogen sites (Solid-liquid ratio = 1:3000 g mL^{-1}, Initial concentration of Th(IV) ≈ 22 mg L^{-1}, pH = 4，T= 25℃).

COFs	$N_{-C=N-}$ content (mmol L^{-1})	N_{Im} content (mmol L^{-1})	Th(IV) concentration (mmol L^{-1})	H^+ concentration (mmol L^{-1})	Th(IV) uptake capacity (mg g^{-1})
Py-TFImI-25	1.28	0	0.1	0.1	43.1
Py-TFIm-25	1.39	0.35	0.1	0.1	61.2

Supplementary Table 6. Analysis of nitrogen sites (Solid-liquid ratio = 1:3000 g mL^{-1}, Initial concentration of Th(IV) ≈ 273 mg L^{-1}, pH = 4，T= 25℃).

COFs	$N_{-C=N-}$ content (mmol L^{-1})	N_{Im} content (mmol L^{-1})	Th(IV) concentration (mmol L^{-1})	H^+ concentration (mmol L^{-1})	Th(IV) uptake capacity (mg g^{-1})
Py-TFImI-25	1.28	0	1.2	0.1	468.3
Py-TFIm-25	1.39	0.35	1.2	0.1	822.6

4. Supplementary Note 1

Since the imidazole nitrogen site would be protonated before the imine nitrogen, we analyzed the occupation of the nitrogen sites at different thorium concentrations. When the initial concentration of Th(IV) is about 22 mg L^{-1} and the pH is 4, the initial concentration is 0.1 mmol L^{-1} for Th(IV) and 0.1 mmol L^{-1} for H^+, while the content of imidazole nitrogen (N_{Im}) is 0.35 mmol L^{-1} (Supplementary Table 5). Moreover, the hydrogen ions will not be completely bound to imidazole nitrogen sites due to the presence of protonation constant. Therefore, at this concentration, the main adsorption site of Py-TFIm-25 COF is the imidazole nitrogen, while the adsorption site of Py-TFImI-25 COF is the imine nitrogen.

When the initial Th(IV) concentration increased 12 times, the uptake capacity of Py-TFImI-25 COF for Th(IV) increased by 10.9 times and the uptake capacity of Py-TFIm-25 COF for Th(IV) increased by 13.4 times (Supplementary Table 5 and 6). In this adsorption system, there are hydrolysis equilibriums of thorium ions, proton equilibriums, and reaction equilibriums. When the pH is fixed, the difference between Py-TFImI-25 COF and Py-TFIm-25 COF adsorption systems should focus on the difference of the reaction equilibrium constants, k_1 for the imine site and k_2 for the imidazole site. The 10.9-fold increase in the uptake capacity of Py-TFImI-25 COF is due to the contribution of k_1, while the 13.4-fold increase in the uptake capacity of Py-TFIm-25 COF is due to the combined contribution of k_1 and k_2. Therefore, at higher concentrations, the contribution of imidazole nitrogen cannot be neglected.

5. References

1 Auras, F. *et al.* Synchronized Offset Stacking: A Concept for Growing Large-Domain and Highly Crystalline 2D Covalent Organic Frameworks. *J. Am. Chem. Soc.* **138**, 16703-16710, doi:10.1021/jacs.6b09787 (2016).

2 Huang, N., Wang, P., Addicoat, M. A., Heine, T. & Jiang, D. L. Ionic Covalent Organic Frameworks: Design of a Charged Interface Aligned on 1D Channel Walls and Its Unusual Electrostatic Functions. *Angew. Chem. Int. Ed.* **56**, 4982-4986, doi:10.1002/anie.201611542 (2017).

3 Chai, Y. Y. et al. N-Heterocyclic Carbene Functionalized Covalent Organic Framework for Transesterification of Glycerol with Dialkyl Carbonates. *Catalysts* 11, doi:10.3390/catal11040423 (2021).

4 Yan, Q. et al. Immobilization of Ionic Liquid on a Covalent Organic Framework for Effectively Catalyzing Cycloaddition of CO_2 to Epoxides. *Molecules* **27**, 6204 doi:10.3390/molecules27196204 (2022).

5 Teixeira, F. C., Rangel, C. & Teixeira, A. New azaheterocyclic aromatic diphosphonates for hybrid materials for fuel cell applications. *New J. Chem.* 37, 3084-3091 doi:10.1039/C3NJ00585B (2013).

6 Krack, M. & Parrinello, M. All-electron ab-initio molecular dynamics. *PCCP* **2**, 2105-2112, doi:10.1039/B001167N (2000).

7 Kühne, T. D. *et al.* CP2K: An electronic structure and molecular dynamics software package-Quickstep: Efficient and accurate electronic structure calculations. *The Journal of Chemical Physics* **152**, 194103, doi:10.1063/5.0007045 (2020).

8 VandeVondele, J. *et al.* Quickstep: Fast and accurate density functional calculations using a mixed Gaussian and plane waves approach. *CoPhC* **167**, 103-128, doi:10.1016/j.cpc.2004.12.014 (2005).

9 Goedecker, S., Teter, M. & Hutter, J. Separable dual-space Gaussian pseudopotentials. *PhRvB* **54**, 1703-1710, doi:10.1103/PhysRevB.54.1703 (1996).

10 Hartwigsen, C., Goedecker, S. & Hutter, J. Relativistic separable dual-space Gaussian pseudopotentials from H to Rn. *PhRvB* **58**, 3641-3662, doi:10.1103/PhysRevB.58.3641 (1998).

11 VandeVondele, J. & Hutter, J. Gaussian basis sets for accurate calculations on molecular systems in gas and condensed phases. *J. Chem. Phys.* **127**, 114105, doi:10.1063/1.2770708 (2007).

12 Perdew, J. P., Burke, K. & Ernzerhof, M. Generalized Gradient Approximation Made Simple. *PhRvL* **77**, 3865-3868, doi:10.1103/PhysRevLett.77.3865 (1996).

13 Grimme, S., Antony, J., Ehrlich, S. & Krieg, H. A consistent and accurate ab initio parametrization of density functional dispersion correction (DFT-D) for the 94 elements H-Pu. *J. Chem. Phys.* **132**, 154104, doi:10.1063/1.3382344 (2010).

14 Fattebert, J.-L. & Gygi, F. Density functional theory for efficient ab initio molecular dynamics simulations in solution. *J. Comput. Chem.* **23**, 662-666, doi:10.1002/jcc.10069 (2002).

15 Andreussi, O., Dabo, I. & Marzari, N. Revised self-consistent continuum solvation in electronic-structure calculations. *J. Chem. Phys.* **136**, 064102, doi:10.1063/1.3676407 (2012).

16 Yin, W.-J., Krack, M., Li, X., Chen, L.-Z. & Liu, L.-M. Periodic continuum solvation model integrated with first-principles calculations for solid surfaces. *PROG NAT SCI-MATER* **27**, 283-288, doi:10.1016/j.pnsc.2017.03.003 (2017).

17 Wang, J. *et al.* Effect of pH, ionic strength, humic substances and temperature on the sorption of Th (IV) onto NKF-6 zeolite. *J RADIOANAL NUCL CH* **310**, 597-609, doi:10.1007/s10967-016-4868-4 (2016).

18 Yuan, D. *et al.* Highly efficacious entrapment of Th (IV) and U (VI) from rare earth elements in concentrated nitric acid solution using a phosphonic acid functionalized porous organic polymer adsorbent. *Sep. Purif. Technol.* **237**, doi:10.1016/j.seppur.2019.116379 (2020).

19 Dolatyari, L., Yaftian, M. R. & Rostamnia, S. Adsorption characteristics of Eu (III) and Th (IV) ions onto modified mesoporous silica SBA-15 materials. *Journal of the Taiwan Institute of Chemical Engineers* **60**, 174-184, doi:10.1016/j.jtice.2015.11.004 (2016).

20 Pan, N., Deng, J., Guan, D., Jin, Y. & Xia, C. Adsorption characteristics of Th (IV) ions on reduced graphene oxide from aqueous solutions. *ApSS* **287**, 478-483, doi:10.1016/j.apsusc.2013.10.004 (2013).

21 Guo, X. G. *et al.* Selective Th(iv) capture from a new metal-organic framework with O(-) groups. *Dalton Trans* **49**, 4060-4066, doi:10.1039/c9dt04912f (2020).

22 Anirudhan, T. S. & Rejeena, S. R. Thorium(IV) Removal and Recovery from Aqueous Solutions using Tannin-Modified Poly(glycidylmethacrylate)-Grafted Zirconium oxide Densified Cellulose. *Ind. Eng. Chem. Res.* **50**, 13288-13298, doi:10.1021/ie2015679 (2011).

23 Alqadami, A. A., Naushad, M., Alothman, Z. A. & Ghfar, A. A. Novel metal–organic framework (MOF) based composite material for the sequestration of U (VI) and Th (IV) metal ions from aqueous environment. *ACS Appl. Mater. Inter.* **9**, 36026-36037, doi:10.1021/acsami.7b10768 (2017).

24 Xiong, X. H. *et al.* Selective extraction of thorium from uranium and rare earth elements using sulfonated covalent organic framework and its membrane derivate. *Chem. Eng. J.* **384**, doi:10.1016/j.pmatsci.2019.01.005 (2020).

25 Liu, X. *et al.* Selective entrapment of thorium using a three-dimensional covalent organic framework and its interaction mechanism study. *Sep. Purif. Technol.* **296**, 121413, doi:10.1016/j.seppur.2022.121413 (2022).
